# Supplementary figures and images for: Geochemical exploration of rare earth element resources in highland karstic bauxite deposits in the Sierra de Bahoruco, Pedernales Province, Southwestern Dominican Republic
Source: PLoS One. 2025 Jan 10;20(1):e0315147. doi: 10.1371/journal.pone.0315147 (PMC11723596; doi:10.1371/journal.pone.0315147)

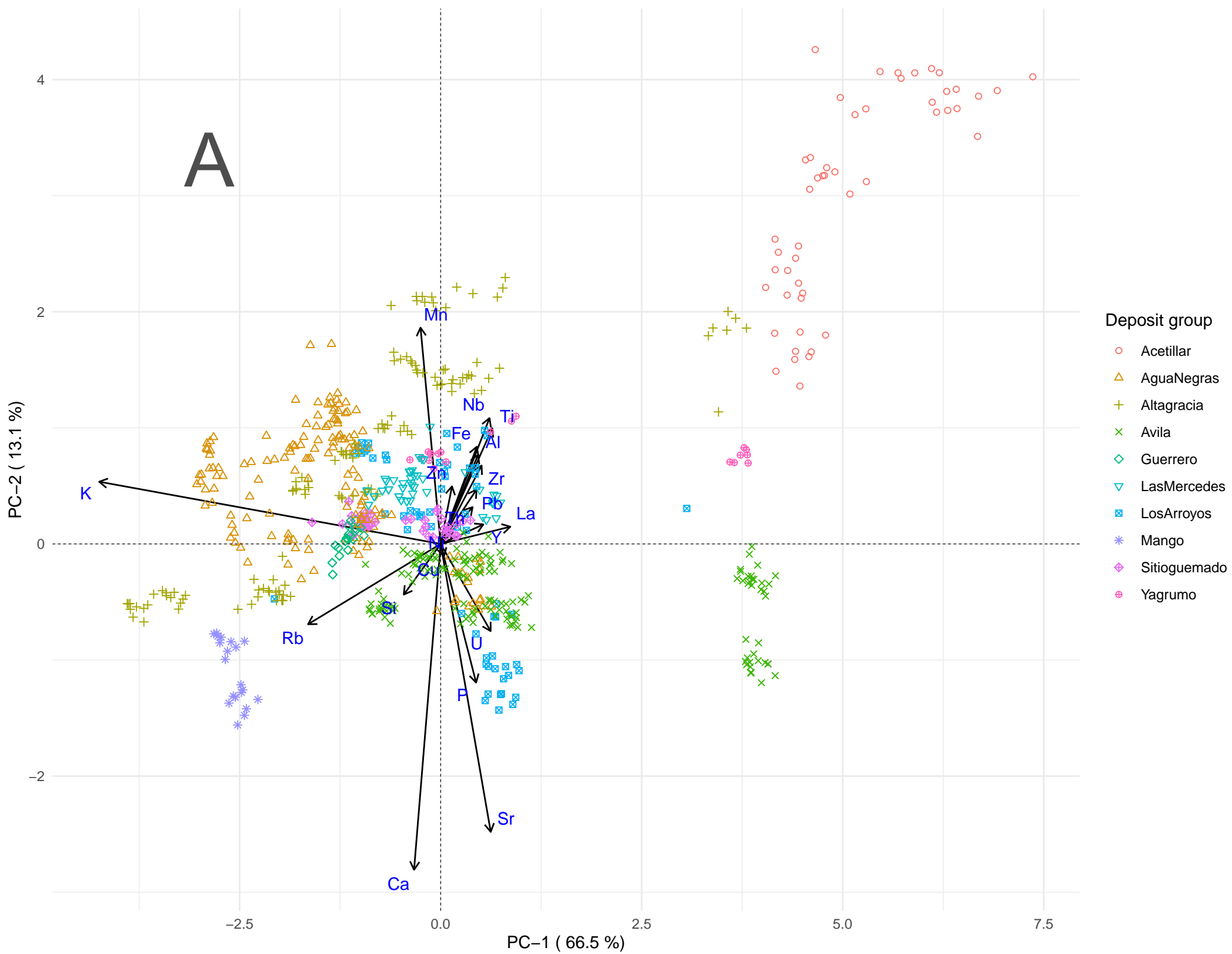

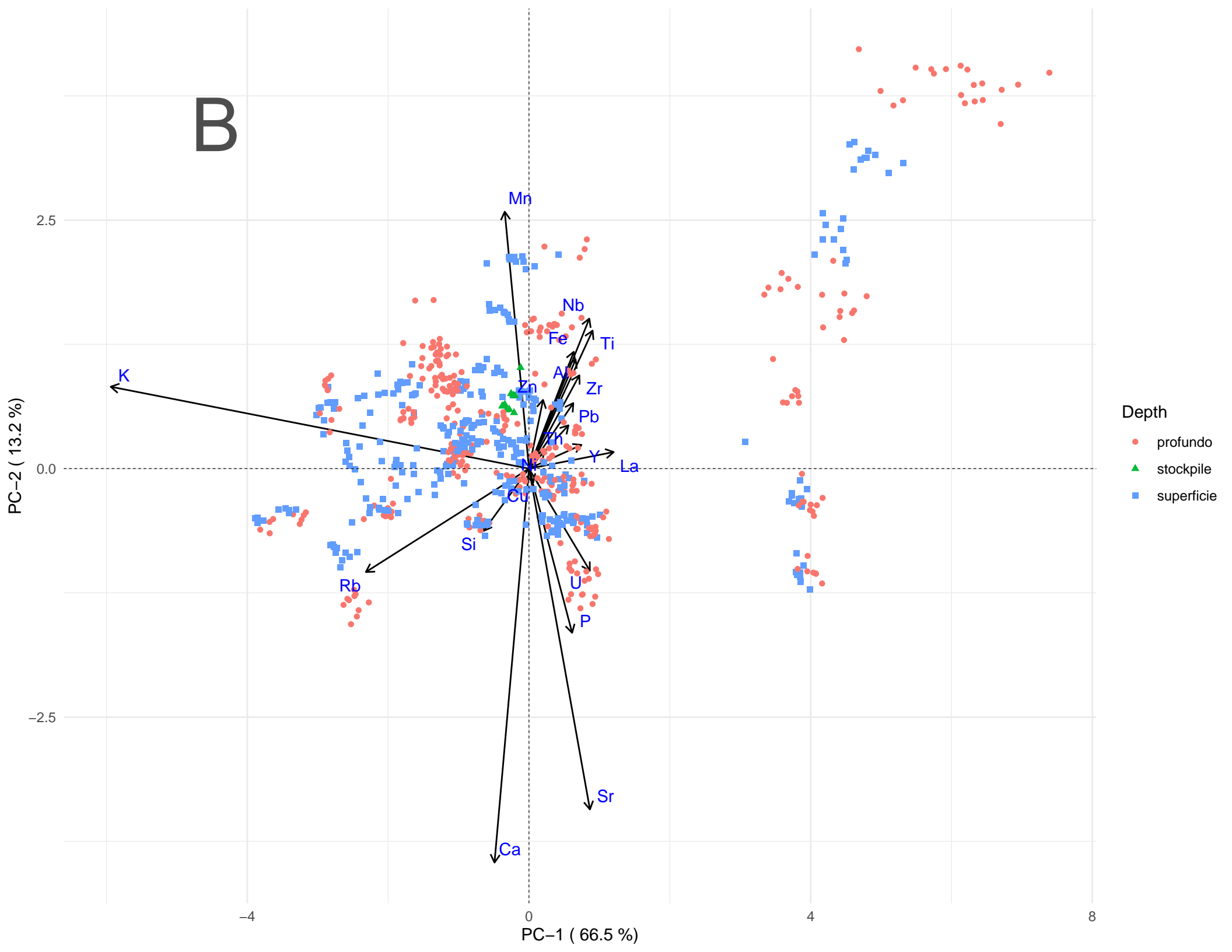

Supplement: S1 Fig — Samples were group based on (A) deposit and (B) depth of collection (where superficie = surface and profundo = subsurface). (PDF) [file pone.0315147.s001.pdf]
