# Supplementary material for: Geochemical exploration of rare earth element resources in highland karstic bauxite deposits in the Sierra de Bahoruco, Pedernales Province, Southwestern Dominican Republic
Source: PLoS One. 2025 Jan 10;20(1):e0315147. doi: 10.1371/journal.pone.0315147 (PMC11723596; doi:10.1371/journal.pone.0315147)
Supplement: S2 Fig — (A) Example statistical distribution testing between two different bauxite deposit groups. (B) Preliminary multidimensional scaling (MDS) results showing the statistical clustering of the different deposit groupings. (PDF) [file pone.0315147.s002.pdf]

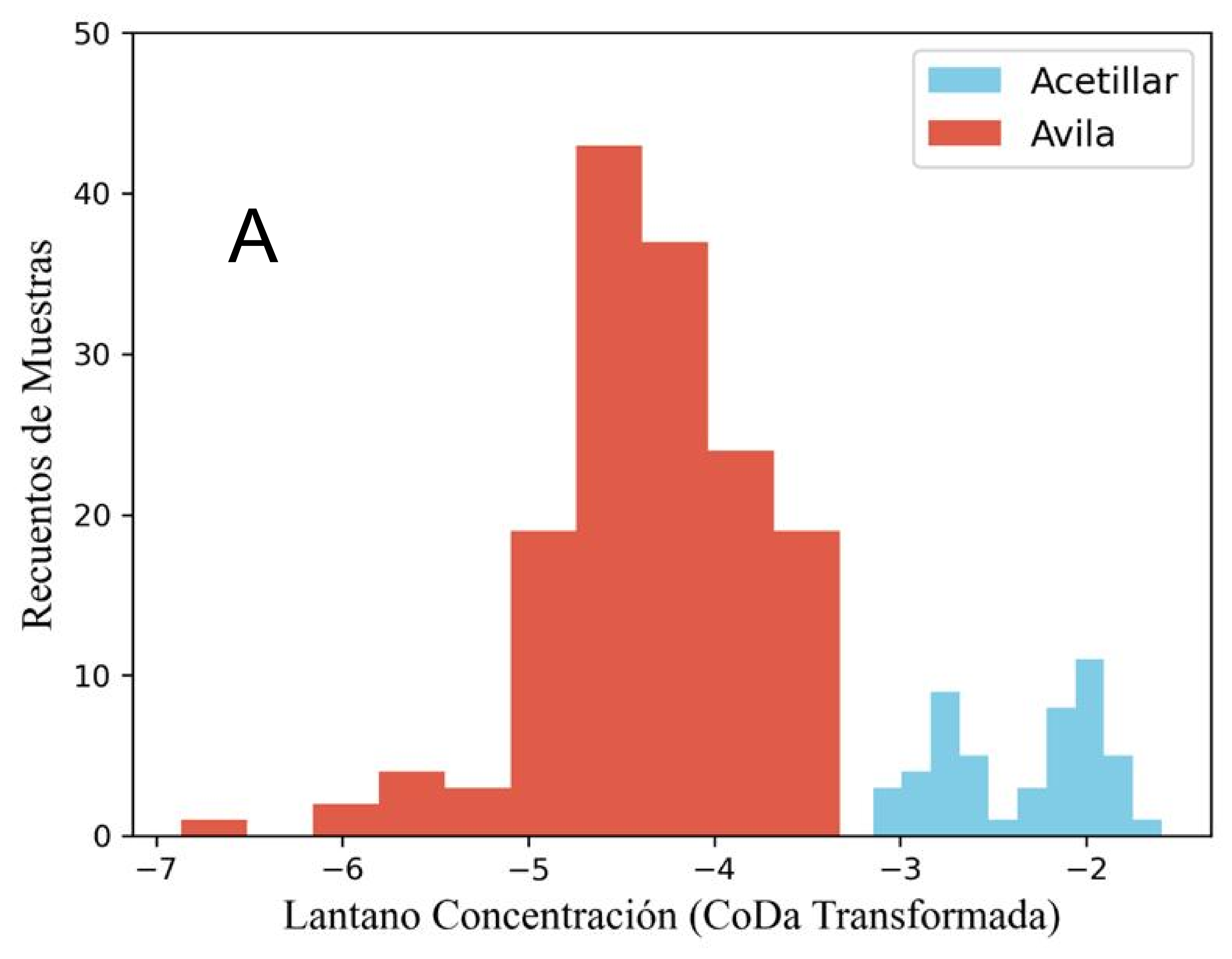

A
Acetillar
Avila
Recuentos de Muestras
Lantano Concentración (CoDa Transformada)
0
10
20
30
40
50
−7
−6
−5
−4
−3
−2

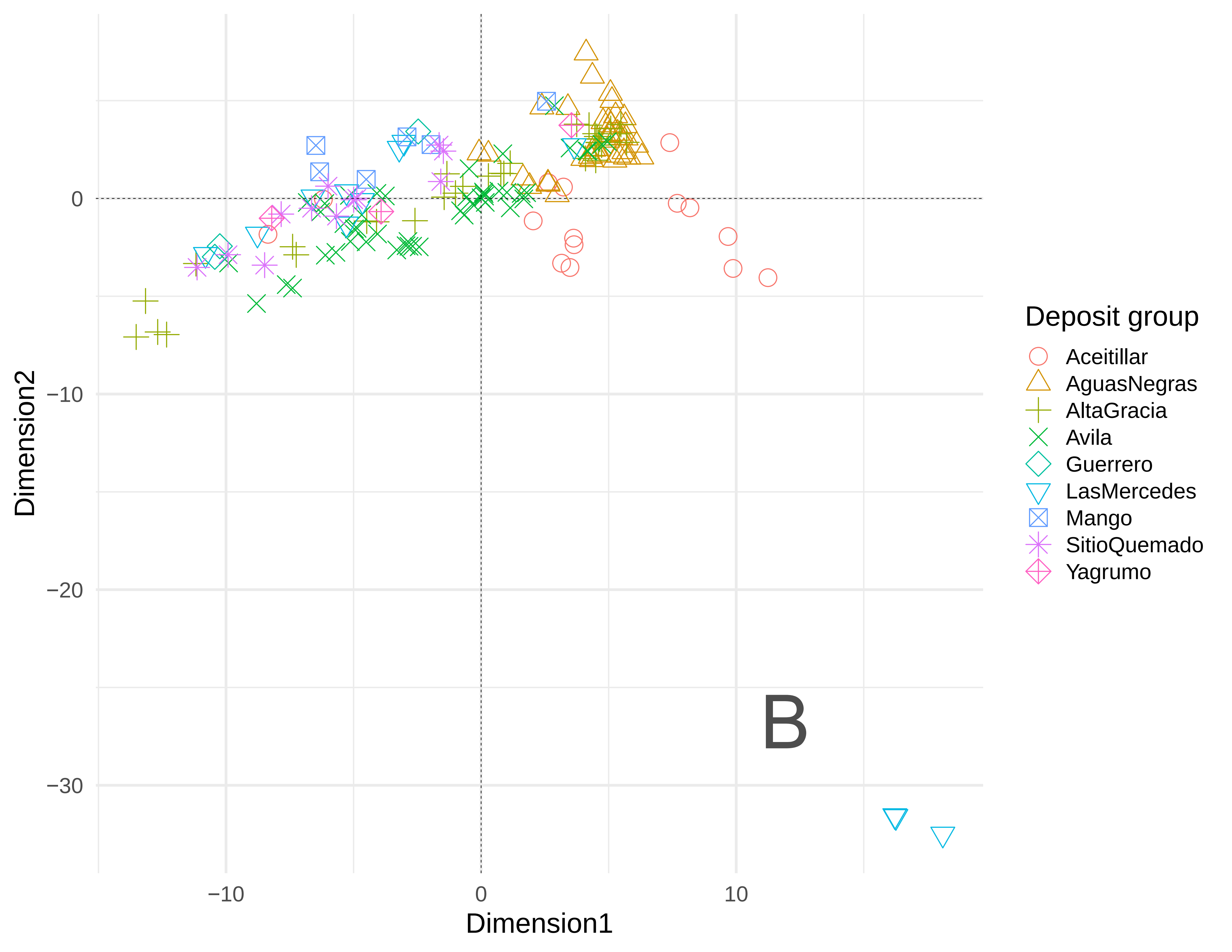

Dimension2
Dimension1
0
−10
−20
−30
−10
0
10
B
Deposit group
Aceitillar
AguasNegras
AltaGracia
Avila
Guerrero
LasMercedes
Mango
SitioQuemado
Yagrumo

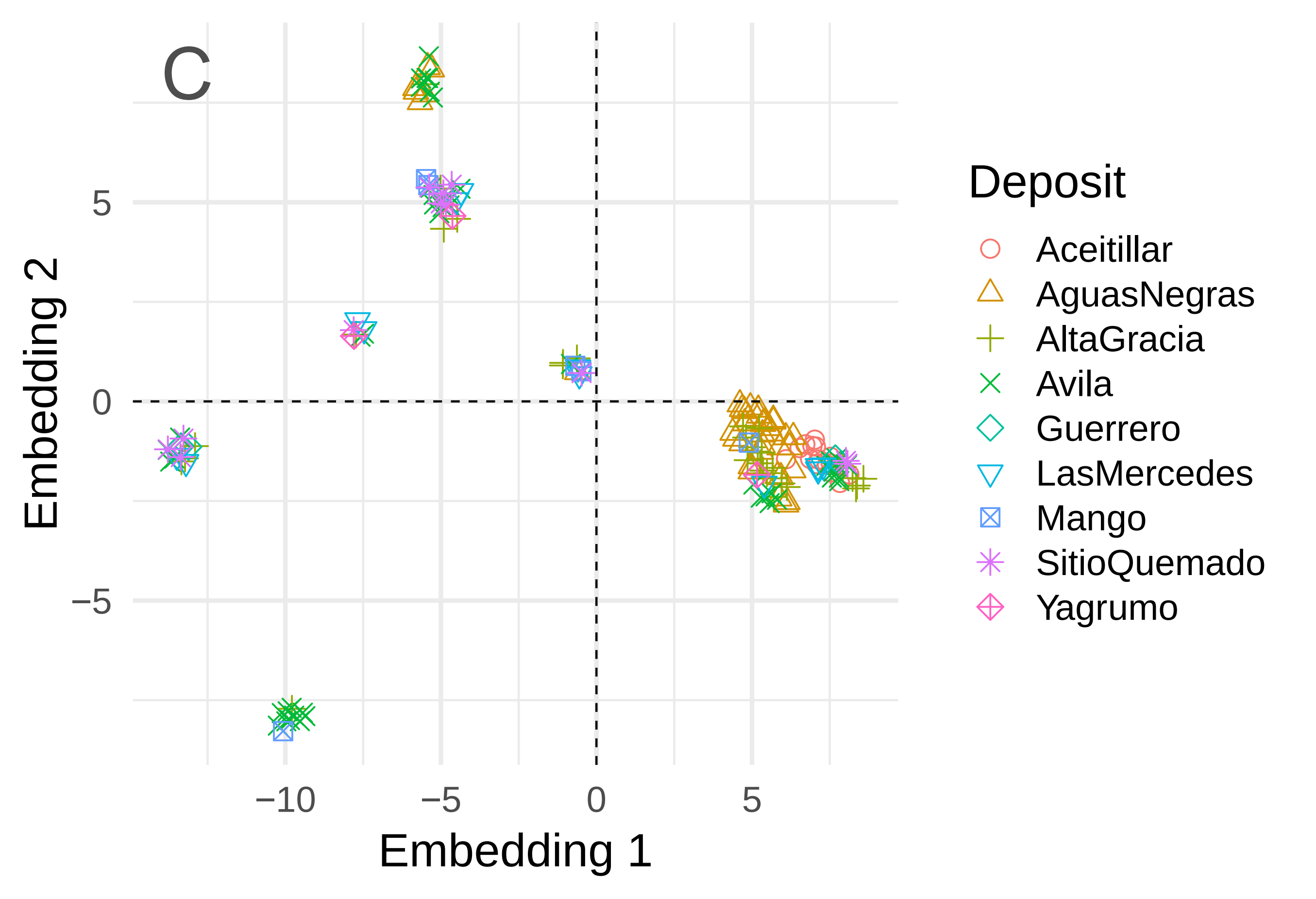
C
Embedding 2
Embedding 1
5
0
−5
−10
−5
0
5
Deposit
Aceitillar
AguasNegras
AltaGracia
Avila
Guerrero
LasMercedes
Mango
SitioQuemado
Yagrumo
