# Supplementary material for: Geochemical exploration of rare earth element resources in highland karstic bauxite deposits in the Sierra de Bahoruco, Pedernales Province, Southwestern Dominican Republic
Source: PLoS One. 2025 Jan 10;20(1):e0315147. doi: 10.1371/journal.pone.0315147 (PMC11723596; doi:10.1371/journal.pone.0315147)
Supplement: S1 File — (DOCX) [file pone.0315147.s003.docx]

# Supporting Information

Geochemical exploration of rare earth element resources in highland karstic bauxite deposits in the Sierra de Bahoruco, Pedernales Province, Southwestern Dominican Republic

Mark Chappell^1^*, Harold Rojas^2^, Charles Andros^1^, Autumn Acree^1^, Yoko Masue-Slowey^1^, Christine Young^1^, Paige Fowler^1^, Wesley Rowland^1^, Michelle Wynter^1^, and Leopoldo Gonzalez^2^

*Corresponding author: mark.a.chappell@usace.army.mil

^1^ Environmental Laboratory, U.S. Army Engineer Research & Development Center, Vicksburg, Mississippi, United States of America,

^2^ Dirección general de minería, República Dominicana

## Cluster analysis for pXRF data.

Early in this study, we utilized different approaches to study the cluster analysis on the pXRF data. Most approaches created unsatisfactory, providing fairly ambiguous groupings of the data. Fig. S1 contains example of using a robust CoDA-transformed principal component analysis (PCA). As an unsupervised method, we attempted to find patterns by labeling the data with respect to deposit type and sample depth.

**S1 Fig. Biplots of generated from a robust PCA for the CoDA-transformed pXRF data.** Samples were group based on (A) deposit and (B) depth of collection (where superficie = surface and profundo = subsurface).

We further attempted a cluster analysis using the pXRF composition, which we hoped would facilitate REE prospecting efforts using only the Niton XL5 by relating zone similarity to ∑REE. To perform the cluster analysis, distributions of the CoDa-transformed La concentrations were prepared for each bauxite deposit zone. Because many of these distributions were non-normal (according to the Shapiro-Wilk test), we used the pair-wise Wilcoxon Signed Rank test to determine the similarity of La concentration among the bauxites. We then created a binary distance matrix to relate each bauxite zone to another by measuring whether the La concentration population means for the two zones were similar (1) or not (0). Finally, we visualized this binary distance matrix (Fig. S2) using multidimensional scaling (MDS) on the *clr*-transformed data. Identifying these groups may be beneficial for developing more accurate prediction models, as the model can be tailored to the particular geochemical distinctiveness of each identified bauxite type. The main advantage of such an approach is to support the lower-cost geochemical exploratory techniques, such as pXRF, or even the color sensor-based technique (discussed below).

**S2 Fig. Statistical tests for distinguishing the different bauxite depostis.** (A) Example statistical distribution testing between two different bauxite deposit groups. (B) Preliminary multidimensional scaling (MDS) results showing the statistical clustering of the different deposit groupings. (C) Results of unsupervised UMAP calculation, plotted by Deposit group.
