# Supplementary material for: Geochemical exploration of rare earth element resources in highland karstic bauxite deposits in the Sierra de Bahoruco, Pedernales Province, Southwestern Dominican Republic
Source: PLoS One. 2025 Jan 10;20(1):e0315147. doi: 10.1371/journal.pone.0315147 (PMC11723596; doi:10.1371/journal.pone.0315147)

PLOS ONE

Geochemical exploration of rare earth element resources in highland karstic bauxite deposits in the Sierra de Bahoruco, Pedernales District, Southwestern Dominican Republic

--Manuscript Draft--

**Manuscript Number:**	PONE-D-23-43088
**Article Type:**	Research Article
**Full Title:**	Geochemical exploration of rare earth element resources in highland karstic bauxite deposits in the Sierra de Bahoruco, Pedernales District, Southwestern Dominican Republic
**Short Title:**	Rare earth element resource geochemical exploration in the Dominican Republic
**Corresponding Author:**	Mark A. Chappell<br>US Army Corps of Engineers EL: US Army Engineer Research and Development Center Environmental Laboratory<br>Vicksburg, MS UNITED STATES
**Keywords:**	rare earth elements, geochemical exploration, red bauxite soils
**Abstract:**	The highly weathered bauxite deposits scattered throughout Southwestern Dominican Republic represent a potentially rare earth element (REE) resource. While little is known about these unusual bauxites, their existence as surface deposits (i.e., found in the surface soils throughout the Sierra de Bahoruco) potentially represent an easily accessible REE resource for the nation. Here, we present the results of a first-phase scientific study focused on the geochemical exploration of REEs in karstic bauxite surface soils. We hypothesized that the original REE-bearing minerals were highly weathered; thus, higher REE concentrations tracked with the morphological development of the soil profile. Given the area's remoteness and terrain's ruggedness, the primary geochemical exploration was conducted using portable x-ray fluorescence and a NixPro2 color sensor. Measurements were taken on processed soil samples collected at the surface and then approx. 1 m depths at various sites within the Sierra de Bahoruco highlands. Given the limits of both the portable x-ray fluorescence and NixPro2 technologies, we developed different, compositional data analysis-transformed, decision tree-based machine learning regression models against data obtained from acid-digested, inductively coupled plasma – optical emission spectrsocpy-analyzed bauxite samples to predict the total soil REE concentrations. Overall, REEs distributions tracked with soil morphological development for relatively undisturbed areas, representing an average increase of 90 mg kg-1 with depth, and biased for light REEs similar to the original pyroclastic source. Soil REE concentrations were significantly and positively correlated with sample latitude and elevation, suggesting that exploration higher up the mountain could yield deposits potentially richer in REEs. A high-quality machine learning model was developed from the x-ray fluorescence, while the color sensor gave a less accurate model due to its limited wavelength range. Overall, we found that portable x-ray fluorescence represented a viable option for remote geochemical exploration of REEs when rigorously calibrated to overcome the instrument's limitations.
**Order of Authors:**	Mark A. Chappell, Ph.D.
	Harold Rojas, B.S.
	Charles Andros, M.S.
	Autumn Acree, Ph.D.
	Yoko Slowey, Ph.D.
	Marcelo Salles, B.S.
	Christine Young, M.S.
	Paige Fowler, M.S.
	Michelle Wynter, Ph.D.

Powered by Editorial Manager® and ProduXion Manager® from Aries Systems Corporation

	Leopoldo Gonzalez
**Additional Information:**	
**Question**	**Response**
**Financial Disclosure**<br><br>Enter a financial disclosure statement that describes the sources of funding for the work included in this submission. Review the submission guidelines for detailed requirements. View published research articles from *PLOS ONE* for specific examples.<br><br>This statement is required for submission and **will appear in the published article** if the submission is accepted. Please make sure it is accurate.<br><br>**Funded studies**<br>Enter a statement with the following details:<br>• Initials of the authors who received each award<br>• Grant numbers awarded to each author<br>• The full name of each funder<br>• URL of each funder website<br>• Did the sponsors or funders play any role in the study design, data collection and analysis, decision to publish, or preparation of the manuscript?<br><br>Did you receive funding for this work?	The author(s) received no specific funding for this work.
**Competing Interests**<br><br>Use the instructions below to enter a competing interest statement for this submission. On behalf of all authors, disclose any competing interests that could be perceived to bias this work—acknowledging all financial support and any other relevant financial or non-financial competing interests.<br><br>This statement is required for submission and **will appear in the published article** if the submission is accepted. Please make sure it is accurate and that any funding	The authors have declared that no competing interests exist.

Powered by Editorial Manager® and ProduXion Manager® from Aries Systems Corporation

sources listed in your Funding Information later in the submission form are also declared in your Financial Disclosure statement.<br><br>View published research articles from *PLOS ONE* for specific examples.<br><br>**NO authors have competing interests**<br><br>Enter: *The authors have declared that no competing interests exist.*<br><br>**Authors with competing interests**<br><br>Enter competing interest details beginning with this statement:<br><br>*I have read the journal's policy and the authors of this manuscript have the following competing interests: [insert competing interests here]*<br><br>* typeset	
**Ethics Statement**<br><br>Enter an ethics statement for this submission. This statement is required if the study involved:<br><br>• Human participants<br>• Human specimens or tissue<br>• Vertebrate animals or cephalopods<br>• Vertebrate embryos or tissues<br>• Field research<br><br>Write "N/A" if the submission does not require an ethics statement.<br><br>General guidance is provided below. Consult the submission guidelines for detailed instructions. **Make sure that all information entered here is included in the Methods section of the manuscript.**	N/A

**Format for specific study types**<br><br>**Human Subject Research (involving human participants and/or tissue)**<br>• Give the name of the institutional review board or ethics committee that approved the study<br>• Include the approval number and/or a statement indicating approval of this research<br>• Indicate the form of consent obtained (written/oral) or the reason that consent was not obtained (e.g. the data were analyzed anonymously)<br><br>**Animal Research (involving vertebrate animals, embryos or tissues)**<br>• Provide the name of the Institutional Animal Care and Use Committee (IACUC) or other relevant ethics board that reviewed the study protocol, and indicate whether they approved this research or granted a formal waiver of ethical approval<br>• Include an approval number if one was obtained<br>• If the study involved *non-human primates*, add *additional details* about animal welfare and steps taken to ameliorate suffering<br>• If anesthesia, euthanasia, or any kind of animal sacrifice is part of the study, include briefly which substances and/or methods were applied<br><br>**Field Research**<br><br>Include the following details if this study involves the collection of plant, animal, or other materials from a natural setting:<br>• Field permit number<br>• Name of the institution or relevant body that granted permission	
**Data Availability**<br><br>Authors are required to make all data underlying the findings described fully available, without restriction, and from the time of publication. PLOS allows rare exceptions to address legal and ethical concerns. See the PLOS Data Policy and FAQ for detailed information.	Yes - all data are fully available without restriction

Powered by Editorial Manager® and ProduXion Manager® from Aries Systems Corporation

A Data Availability Statement describing where the data can be found is required at submission. Your answers to this question constitute the Data Availability Statement and **will be published in the article**, if accepted.<br><br>**Important:** Stating 'data available on request from the author' is not sufficient. If your data are only available upon request, select 'No' for the first question and explain your exceptional situation in the text box.<br><br>Do the authors confirm that all data underlying the findings described in their manuscript are fully available without restriction?	
**Describe where the data may be found in full sentences. If you are copying our sample text, replace any instances of XXX with the appropriate details.**<br><br>• If the data are **held or will be held in a public repository**, include URLs, accession numbers or DOIs. If this information will only be available after acceptance, indicate this by ticking the box below. For example: *All XXX files are available from the XXX database (accession number(s) XXX, XXX.).*<br>• If the data are all contained **within the manuscript and/or Supporting Information files**, enter the following: *All relevant data are within the manuscript and its Supporting Information files.*<br>• If neither of these applies but you are able to provide **details of access elsewhere**, with or without limitations, please do so. For example:<br><br>*Data cannot be shared publicly because of [XXX]. Data are available from the XXX Institutional Data Access / Ethics Committee (contact via XXX) for researchers who meet the criteria for access to confidential data.*<br><br>*The data underlying the results presented in the study are available from (include the name of the third party*	Data is available upon request

and contact information or URL).<br>• This text is appropriate if the data are owned by a third party and authors do not have permission to share the data.<br><br>* typeset	
Additional data availability information:	

Powered by Editorial Manager® and ProduXion Manager® from Aries Systems Corporation

Manuscript Click here to access/download;Manuscript;REE paper_16Jan2024.docx

Full title: Geochemical exploration of rare earth element resources in highland karstic bauxite deposits in the Sierra de Bahoruco, Pedernales District, Southwestern Dominican Republic

Short title: Rare earth element resource geochemical exploration in the Dominican Republic.

Mark Chappell[1]*, Harold Rojas[2], Charles Andros[1], Autumn Acree[1], Yoko Slowey[1], Marcelo Salles[3], Christine Young[1], Paige Fowler[1], Wesley Rowland[1], Michelle Wynter[1], and Leopoldo Gonzalez[2]

*Corresponding author: mark.a.chappell@usace.army.mil

[1] Environmental Laboratory, U.S. Army Engineer Research & Development Center, Vicksburg, Mississippi, United States of America,

[2] Dirección general de minería, República Dominicana

[3] South and Central America and Caribbean Region, South Atlantic Division, U.S. Army Corps of Engineers, United States of America

Abstract

the abstarct is too long. the objectives are not clear enough to the general reader

The highly weathered bauxite deposits scattered throughout Southwestern Dominican Republic represent a potentially rare earth element (REE) resource. While little is known about these unusual bauxites, their existence as surface deposits (i.e., found in the surface soils throughout the Sierra de Bahoruco) potentially represent an easily accessible REE resource for the nation. Here, we present the results of a first-phase scientific study focused on the geochemical exploration of REEs in karstic bauxite surface soils. We hypothesized that the original REE-bearing minerals were highly weathered; thus, higher REE concentrations tracked with the morphological development of the soil profile. Given the area's remoteness and terrain's ruggedness, the primary geochemical exploration was conducted using portable x-ray fluorescence and a NixPro2 color sensor. Measurements were taken on processed soil samples collected at the surface and then

add GPS INFORMATION

approx. 1 m depths at various sites within the Sierra de Bahoruco highlands. Given the limits of both the portable x-ray fluorescence and NixPro2 technologies, we developed different, compositional data analysis-transformed, decision tree-based machine learning regression models against data obtained from acid-digested, inductively coupled plasma – optical emission spectrsocpy-analyzed bauxite samples to predict the total soil REE concentrations. Overall, REEs distributions tracked with soil morphological development for relatively undisturbed areas, representing an average increase of 90 mg kg^{-1} with depth, and biased for light REEs similar to the original pyroclastic source. Soil REE concentrations were significantly and positively correlated with sample latitude and elevation, suggesting that exploration higher up the mountain could yield deposits potentially richer in REEs. A high-quality machine learning model was developed from the x-ray fluorescence, while the color sensor gave a less accurate model due to its limited wavelength range. Overall, we found that portable x-ray fluorescence represented a viable option

for remote geochemical exploration of REEs when rigorously calibrated to overcome the instrument's limitations.

Introduction

you use REE above and here you use REEs . this must be the same

Rare earth elements (REEs) represent 15 lanthanides with atomic numbers 57 to 71 in the periodic table, as well as scandium (Sc), yttrium (Y), and lanthanum (La) representing atomic numbers 21, 39, and 57, respectively. REEs are critical components of modern technologies and are important for a variety of industrial, medical, and particularly new "green-energy" applications. General REE applications include radar and sonar applications, communications and heads-up displays (HUDs), high-capacity electric motors, and jet engines (Trytek, 2019, and references therein). For example, neodymium (Nd) alloys for the basis for high-performance magnets used wind turbines and electric vehicles – its use requiring less mass for an equivalently performing generator or electric engine, such as traditional Fe-based electromagnets (Sprecher et al., 2014, and references therein). The ability for Cerium (Ce), the most common of the REEs, can not only serve as a "hard" cationic replacement for Ca^{2+} for anticoagulant/anti-clotting agents, but is important for the catalytic scavenging of O and S for improving alloy oxidative resistance (Dahle and Arai, 2015). The hydride form of the REE yttrium (Y) is currently being explored for its ability to serve as moderators for portable, micro- or small-scale nuclear reactors (Hu et al., 2020), under development for emergency power generation, such as after civilian infrastructural destruction following substantial natural disaster events. Dysprosium (Dy) and terbium (Tb) are vital for the production of capacitors, batteries, and superconductors used in radar systems, communication devices, and sonar equipment (Hedrick, 2004). Laser systems employ Nd-doped Y-Al garnet (Nd YAG) in many range-finding applications (Mancheri et al., 2013).

Despite their label, REEs are moderately abundant (except for promethium or Pm) and only considered "rare" given their comparatively low quantities relative to locations that support economic mining (Leal Filho et al., 2023). Traditionally, economically viable REE concentrations exist in carbonatites, alkaline igneous settings, ion-adsorption clays, and monazite-xenotime-bearing placer deposits due to deep weathering and fluvial processes (Borst et al., 2020; Leal Filho et al., 2023). However, for this work, we were interested in exploring REE soil concentrations and distribution in a less traditional setting - highly weathered karstic bauxite deposits within the Sierra de Bahoruco Mountains in the Pedernales Province located in Southwestern Dominican Republic (DR). Regionally, these deposits represent a potentially strategic source of REEs. The bauxites are believed to originate from either one of two sources: Ash deposited from explosive Miocene volcanic eruptions in Central America that occurred some 20M years ago (Goldich, 1947; Grant et al., 2005) or paleo-erosional material formation resulting from post-Pliocene geological uplift of the Bahoruco Peninsula (Villanova-de-Benavent et al., 2017; Villanova-de-Benavent et al., 2023) from igneous source of mafic composition (Escuder-Viruete et al., 2016; Torró et al., 2017). While limited, the available geochemical information points to similarities in the bauxite chemical composition of Jamacia and the Southwestern corner of Hispaniola (Fig. 1), including the DR and Haiti (Goldich, 1947; Grant et al., 2005). Previous studies (Torró et al., 2017; Villanova-de-Benavent et al., 2017; Villanova-de-Benavent et al., 2023) first identified REEs in the inactive bauxite mining site, Las Mercedes, and later at an additional inactive mining site in Aceitillar and separately a location named El Turco. Using hydro-and magnetic separation methods, the authors showed the trace presence of REE-bearing carbonate and phosphate minerals, representing the probable original form of the REEs before millions of years of geochemical weathering.

Fig 1. Theoretical origin of the bauxite deposits is associated with ash deposition from ancient volcanic activity in Central America. The red dashed lines demarcate the proposed zone of ash deposition, where the bauxite deposits exhibit geochemical similarities (adapted from Goldich, 1947).

We hypothesize that intense geochemical weathering from the local climate concentrated these recalcitrant elements relative to average background concentrations post-deposition. LeBlanc et al. (2017) summarized the DR's overall national climate as class A (tropical rainy climates) on the Koppen (1936) scale, based on its monthly mean air temperature > 18 °C and annual mean rainfall amounts. While overall hot and humid climate, there is substantial local variability, attributed mainly to the Cordillera Central mountain range that trends diagonally across Hispaniola, blocking the onshore flow of humid ocean air masses from the northeastern trade winds and creating a strong rain shadow effect, and aridity in the southwestern portion of the country (LeBlanc et al., 2017), particularly around the Pedernales area. Thus, the island's weather and climate, influenced by the mountain chains aligned perpendicular to the prevailing eastern currents, create substantial mean annual rainfall differences between the northeast and southwest of the Cordillera Central mountains. The low-elevation coastal areas, particularly within the Bahoruco Peninsula (and around the town of Pedernales) in the southwestern portion of the country, receive 35 – 40 cm of rainfall, falling within the Koppen category BShw (warm, arid steppe climate) consistent with an arid environment. However, the highland areas of the Sierra de Bahoruco located some 10-15 km north of Pedernales, transition to the Koppen classification Af (LeBlanc et al., 2017), representing a tropical rain forest climate at approx. 1220 m elevation. Apparently, this climate has persisted for millennia. For example, Taggart (2006, and references therein) reported that the Western area of the DR has been historically hot and humic, achieving a

thermal maximum, with the highest temperatures and peak precipitation approx. 6000 years ago, based on delta ^{18}O values for bivalves. Historically, the DR also gains much of its annual moisture through hurricane events, which are strongest during the La Niña phase of the El Niño Seasonal Oscillation (ENSO)

Given that the bauxites are all located at the surface in the gentler slopes and inclines throughout the Sierra de Barhoruco, we approached the REE exploration from a Soil Science perspective instead of the classical geological perspective. We hypothesized that the sustained, intense geochemical weathering of the volcanic ash/ancient erosional/paleosol material had broken a significant proportion of the mineral monzonite, carbonate, and phosphate forms containing REEs. As "free" ions, these REEs would be primarily retained in the soils by reversible cation exchange reactions and evidenced by elevated REE concentrations existing in the heavier subsurface clay regions of the soils (Aide and Aide, 2012; Borst et al., 2020). Clay accumulation in the subsurface layers and argillic horizon development would be consistent with classical soil development and the translocation of finer-sized particles to the soil subsurface. REE-bearing minerals in the soils would not necessarily be expected to conform to this pattern.

The Sierra de Bahoruco exists in the very remote corner of the Dominican Republic, along the southwestern border with Haiti. Here, conducting the geochemical exploration in the traditional sense of a dedicated laboratory is challenging. Logistical, infrastructural, and technical challenges abound in this particular area. It is possible to transport samples to developed regions of the country, such as the capital, Santo Domingo; however, at the time this study was executed, the capital represented a minimum six-hour drive on long stretches of partially developed mountain road that runs directly east of the town of Pedernales to Santo Domingo. In addition, the sheer number of samples required to explore the bauxite deposits would rapidly enhance the operational

expense and uncertainty of the campaign before any analytical work would occur. Thus, opting to focus on a remote exploration scenario, we studied different approaches to measuring the REE concentrations in this expeditionary area. The objectives of this work were to (i) characterize the REE concentrations in the bauxite soils and (ii) explore the use of different calibration approaches to facilitate the geochemical exploration of REEs in remote locations.

Materials & Methods

Field measurements and sampling. Explorations occurred within the Sierra de Bahoruco mountains north of Pedernales, where bauxite samples were collected among several previously identified deposits (Fig. 2A). Among the sampled sites, Aceitillar and Las Mercedes were heavily disturbed due to previous bauxite mining activities. While it was unclear whether bauxite had been removed from the Aceitillar site, it seems that in some areas, as much as 18.3 m of material had been removed at the Las Mercedes site. The remaining sites were less disturbed, mainly through agricultural production. Soil samples were collected at both the surface and subsurface (Fig. 2B), consistent with our hypothesis regarding REE distributions with the soil's natural morphological development and horizon formation. At each location, sampling sites were decided based on the noticeable visual differences between bauxite material and the more calcite-derived soils. For example, the bauxite soils were reddish in appearance and typically free of gravel or large rocks, while the calcite soils were very rocky and usually sparsely vegetated and strongly sloping, characteristic of typical karst topography. The presence of fruit trees and farmland also served as indicators, given the apparent greater fertility of the bauxite soils. For each selected deposit, soils were dug out by hand using a posthole digger and composited from four different holes dug at that deposit to capture some of the site's inherent soil heterogeneity. Soil samples

were collected at the surface and then subsurface - usually representing no more than 30 cm depths, given the presence of a nearly impenetrable dense clay layer. Standard soil and site parameters were determined in the field, including soil color (Munsell color scale), texture (hand test), and, occasionally, effervescence using a 1% HCl solution. In addition, geospatial coordinates, elevation, landscape position, and slope were recorded. However, despite our efforts, we could not assign full NRCS taxonomical designations to the soils given the lack of available annual precipitation data for the region.

Fig 2. (A) Map showing the distribution of sampling sites; (B) Image showing natural horizonation of the bauxite deposits. Samples were collected at the surface and the subsurface, with the strong color change indicating an increase in clay content.

General soil characterization. Collected samples were air-dried, ground, and then sieved (2 mm) in a dedicated laboratory space in Pedernales. Processed samples were shipped to ERDC in Vicksburg, MS, USA, for laboratory analysis. The "bulk" composition (Chappell et al., 2022; Chappell et al., 2019a; Chappell et al., 2019b) of soil elements (including REEs) was determined using a combined microwave-assisted acid digestion, followed by inductively coupled plasma with an Agilent optical emission spectroscopy (ICP-OES). First, 0.5 g of each sample was added to a Teflon tube, then suspended in 3 mL of trace metal grade HCl and 9 mL of trace metal grade nitric acid. Afterward, the samples were digested using a Mars 6 (CEM, Matthews, NC, USA) OneTouch microwave digestor via EPA Method 3051. After digestion, the samples were diluted with approx. 40 mL of water digestates were filtered through 0.45 µm syringe filters and analyzed via an Agilent 5110 ICP-OES. Soil carbon speciation was determined using an Elementar SoliTOC instrument (Elementar Americas, Ronkonkoma, NY, USA).

pXRF analysis. Portable x-ray fluorescence (pXRF) analysis was conducted on processed samples using a Thermo Niton5 XL Plus handheld spectrometer, programmed to measure 44 elements, including 6 REEs. Four different scan levels at 15 s scan times among the four different energy ranges were deemed the most efficient, given energy fluctuations for longer dwell times. The pXRF calibration was tested against REE and NIST 2709a x-ray standards daily to check the instrument's internal calibration. The optimal x-ray fluorescence signal was obtained by placing the spectrometer directly in a plastic weigh boat filled with the powdered sample (Fig. 4). Given the microscale heterogeneity of elemental domains commonly manifested in processed soil samples (Chappell et al., 2022, and references therein), measurements were repeated ten times on each sample, with the detector repositioned at a different place in the weight boat for each measurement. Typical soil elemental and REE concentrations were statistically tested for normal distributions.

Potentiometric measurements. Soil pH, electrical conductivity (EC), and redox potential (Eh) measurements were made using a portable combination probe and battery-operated meter. The probe response was calibrated using proprietary standards.

Soil color measurements. Soil color was determined on the processed soils using a self-calibrating NixPro2 color sensor. Processing the samples was necessary for this determination, given the sensitivity of soil color to moisture. Color measurements were determined by gently pushing the sensor's collection chamber beneath the sample surface and averaging the results of three different scans for different sample positions in the weigh boat.

Computational modeling. Given the compositional nature of soil characterization data, we determined to use compositional data analysis (CoDa) – a technique to correct for geometric

distortions in proportional data (Aitchison, 1986; Aitchison, 1999; Hernández Suárez et al., 2016; Pawlowsky-Glahn and Buccianti, 2011) - for two of these datasets, namely the bulk and the pXRF datasets. Due to the small number of features, the color composition was not processed using CoDA. Data preprocessing was performed using the R statistical computing software (R Development Team, 2018) via the RStudio environment (RStudio Team, 2018). The CoDa workflow dictates that the first step is to remove left-censored data values (e.g., values below the detection limit). Any features comprising more than 5% left-censored data values were removed from the compositions. Any remaining left-censored data values were imputed using the log-ratio Expectation-Maximization (lrEM) algorithm within the zCompositions package (Palarea-Albaladejo and Martin-Fernandez, 2015). Next, each row was closed to a unit sum 100 using the *clo* command (van den Boogaart et al., 2020). Finally, all rows were log-transformed using the additive-log ratio (alr) transformation, normalizing to Si as follows (Eq. 1):

$$alr(x) = \left[\ln\left(\frac{x_1}{x_{Si}}\right), \ldots, \ln\left(\frac{x_{D-1}}{x_{Si}}\right)\right] \qquad [1]$$

where D is the number of components, or features, in the composition. After processing, the bulk composition comprised 25 (D-1) components and 162 samples, the pXRF composition comprised 41 components and 749 samples (considering the ten replicate scans per sample described above), and the color composition comprised three components and 65 samples. Dealing with left-censored data was particularly problematic for the pXRF composition since many elemental concentrations were reported below the detection limit. However, the handheld XRF also reported the uncertainty of its measurements in the form of 2-σ. This uncertainty, we reasoned, represented the maximum value the handheld XRF may have detected. Therefore, the uncertainty values (2 - σ) were added to the detected values to form a new pXRF compositional dataset. The remaining left-censored data were processed using the method described above.

Geochemical clustering was modeled using the nonlinear dimension reduction technique, Uniform Manifold Approximation and Projection or UMAP (McInnes et al., 2018) via the “uwot” package (Melville, 2020) for R. A supervised UMAP model was computed as guided by the assigned sample deposit groups, using n_neighbors and min_dist hyperparameters set at 25 and 0.2, respectively.

Machine learning (ML) modeling was performed using Python3. The model selection was performed by training a suite of candidate regression models using a 3-fold cross-validation on the pXRF composition dataset to predict the sum of REEs or ∑REE. Included were two instance-based models, Support Vector Machine (SVM) and K-Nearest Neighbors (KNN), three tree ensemble models, Random Forest (RF), Extremely Randomized Trees (ET) (Geurts et al., 2006), XGBoost (XGB) (Chen and Guestrin, 2016) and the ordinary least squared (OLS) regression model. Of these models, the SVM, RF, and ET models all performed equally well. As a result, all models were developed using the ET model based on our familiarity with the algorithm in modeling soil geochemistry.

After selecting the ET model, the validation procedure was the same for all three compositions. First, a validation set comprising 15% of the composition’s data was withheld to provide a generalization estimate. Next, resampling was performed by drawing 1,000 different 70% - 15% train-test splits and refitting the ET model at each iteration. Given the spread of values in the target variable ∑REE, binned stratification was employed to represent better the target variable in the randomly selected train-test-validation splits (Rendleman et al., 2022). At the end of the 1,000 iterations, the model with the smallest mean absolute error (MAE) in the target variable was selected and used to predict the held-out validation set. The average MAE for all 1,000 models

was also calculated as a second generalization estimate. The MAE values for each composition are presented in Table 1.

The same modeling procedure was used for the pXRF and the color compositions. Model optimization was performed using a Monte Carlo (MC) simulation. At each step of the MC simulation, a new 66/33 train-test split was taken from the composition of interest, and separate regression models were trained to predict the ICP-OES concentrations for Y, La, and Ce. The simulation was run for 500 steps. At the end of the simulation, the train-test split with the lowest MSE for Y, La, and Ce was selected. ET models were then trained on this optimized split to predict Ce, Dy, Gd, La, Nd, Pr, Sc, and Y. Because the remainder of the REE demonstrated very low concentrations (on average < 30 ppm), the sum of the predicted elements (listed above) was taken as the predicted ∑REE.

Results

Geochemistry of the different deposits

The geochemical signatures of the bauxite deposits were investigated using a compositional diagram of the soil Al, Fe, and Si, as determined from the pXRF data (Fig. 3A) measured on ten different spots within each of the powdered samples. Definitive zones for each of the deposits are apparent in the plot. For example, in terms of deposit type, the Aceitillar, similar to the results of Villanova-de-Benavent et al. (2023), and a portion of the Avila measurements generally fell within the "Bauxite to Fe-rich bauxite" classification proportionally enriched in Al and Si, but relatively low in Fe. Samples from the Los Arroyos deposit spanned both the bauxite and Fe-rich

classifications. Most of the Mango, Aguas Negras, Guerrero, and Sitioquemado measurements clustered in the middle portion of the ternary diagram within the "Clayey bauxite" designation, being proportionally enriched in Al relative to Si, while the majority of the Las Mercedes measurements were classified as "Bauxitic clayey iron ore," being relatively enriched in Al. Most of the Avila, Alta Gracia, and Yagrumo samples were classified as "Bauxtic clays," possessing a relatively low proportion of Si. Replotting the Al-Si-Fe ratios for the NRCS morphological designations (Fig. 3B – note that the heavily disturbed mining sites, Aceitillar and Las Mercedes, were not morphologically characterized and not included in the plot) showed that many of the soils and their heavy clay content qualified them as Vertisols. In general, we observed two main, high-density clusters of Vertisols – in the center of the plot in the area of "Clayey bauxites" and at the bottom of the plot in the area of soils classified as "Bauxite clay." In the plot, substantial overlap can be observed among the Alfisols and Vertisols within the ternary diagram.

Supervised UMAP dimension reduction of the full geochemical dataset, as represented by the bulk composition, was superior for emphasizing local structure (represented by the two-dimensional plot of the embeddings) based on the deposit group labels (Fig. 3C) compared to principal component analysis and multidimensional scaling methods as well as an unsupervised UMAP analysis (see Supporting Information section). From a geochemical perspective, samples within the Aceitillar and AgasNegras deposit groups as well as one triplicate of a LasMercedes sample, mapped out as much more distinguishable clusters based on their deposit group assignments (Fig. 3C). The remaining deposits were less distinguishable by any dimension reduction technique used, suggesting that the deposits group labels require further refinement as many samples located in the middle of the map were geochemically similar. The greatest geochemical discriminator in the

middle portion of the sample area may be associated with elevation and the dramatic shift from a more arid to tropically wet climate within the Sierra de Bahoruco range.

Fig 3. Different visualizations of the geochemical data. Ternary plots showing the relationship among Si, Al, and Fe concentrations (as determined by pXRF analysis of 10 different spots on powderized samples) with respect to (A) Deposit group and (B) Order in the NRCS soil morphological classification system. (C) UMAP decomposition of geochemical data determined by acid-digested, ICP-OES-determined REEs showing the latent structural relationships among the different bauxite deposits.

Soil REE concentrations

The data showed differences in the sum of REE concentrations (plotted in $\log_{10}$ concentrations) between the surface (change the label to "Surface") and subsurface (change plot label to "Subsurface) depths (Fig. 4A, also see the Supporting Information section). For the surface layer, the formerly mined deposit group, Aceitillar possessed the highest median summed REE concentration at 2258 mg total REEs kg^{-1}, with the highest concentration of one sample at 1.3%. Among the deposits located at higher elevations (> 975 m), Los Arroyos possessed the greatest median REE surface concentration of 1325 mg kg^{-1}, which was approx. 400 mg kg^{-1} higher than the median REE concentration at the Avila and Guerrero deposits. In contrast, bauxite soils located in the lower elevation = 610 m) Yagrumo, Mango, Sitio Quemado, and Las Mercedes deposits exhibited the REE median surface concentrations approx. 300 to 400 mg kg^{-1} lower than the higher elevation deposits. In particular, at the heavily impacted Las Mercedes site, the "surface" sample, representing what was left after some 18.3 m of bauxite removal, exhibited a median REE concentration of 630 mg kg^{-1}. For the heavily impacted former mining sites, the median REE subsurface concentration for Las Mercedes was only 5 mg kg^{-1} higher than the surface

concentration. In contrast, the subsurface median REE concentration for the Aceitillar deposit group was half of the surface REE concentration, even though one sample contained 0.25% REEs. For the less-impacted deposits, the higher elevation Los Arroyos, Aguas Negras, and Avila deposits exhibited subsurface median REE concentrations of 555, 245, and 140 mg kg^{-1} greater than the surface median concentrations. The lower elevation Yagrumo deposit increased subsurface median REE concentration by 323 mg kg^{-1}; however, the Mango deposit decreased subsurface median REE concentration by 65 mg kg^{-1}. Overall, the median total REE concentration across the difference deposits was approx. 100 mg kg^{-1} higher in the subsurface than the surface layers. Also, we tracked the ratio of light REEs (Sc, Y, La, Ce, Pr, Nd, Sm) to heavy REEs (Eu, Gd, Tb, Dy, Ho, Er, Tm, Yb, and Lu) and created boxplots to compare their distribution with depth among the different deposits (Fig. 4B, also see the Supporting Information section). For most deposits, there was little change in the median light-to-heavy-REE (LREE/HREE) median ratio with depth. Most deposit groups grew slightly more enriched in HREEs with the 1 m depth, except for Las Mercedes, Mango, and the Sitio Quemado deposits.

Fig 4. Boxplots showing the statistical distributions of the REE data. (A) the measured $\log_{10}$ REE concentration and (B) LREE/HREE ratios in the soils with respect to each particular deposit group for both the surface and subsurface depths.

Given the above trends in the REE distributions, we conducted a correlation analysis (Fig. 5) to search for relationships between the location of the different deposits concerning the total REE concentrations (both surface and subsurface concentrations combined for this analysis). For the analysis, the mining sites Aceitillar and Las Mercedes were removed to diminish the influence of the more disturbed sites on the feature interdependencies. First, the correlation results agree with some basic tenets about the terrain where the study was conducted. For example, there was a

strong and significant correlation (r = 0.96) between the sample latitude and elevation – a sensible result given that the deposits are all distributed on the South-facing mountains (facing the coast), with samples collected at elevations ranging from 233 (the Mango deposit, collected at the base of the Sierra de Bahoruco Mountains) to 1363 m (the Los Arroyos deposit). Our observations and digital maps agreed with the appearance of more abundant and denser vegetative growth with elevation, although exact precipitation totals were unavailable. The results showed a statistically significant positive correlation between total REE concentration and the deposits' terrain elevation (r = 0.337) and latitude (r = 0.356). Generally, higher REE concentrations were measured in the soils developed under a stronger moisture regime. Furthermore, a significant and negative correlation between LREE/HREE ratio elevation (r = -0.355) and latitude (r = -0.277) was observed, indicating that the bauxites become relatively more enriched as the total REE concentrations increased. When the disturbed Aceitillar and Las Mercedes sites were included in the correlation analysis, the results showed a significant relationship between total REE concentration and LREE/HREE with longitude and elevation, artificially pulling on the model (particularly the Aceitillar samples) due to their eastern location in the Sierra de Bahuroco Mountains.

Fig 5. Correlation plot showing the relationships among soil REE concentrations, LREE/HREE ratios, and the geographical positional features of samples collected from the different bauxite deposits. Here, the median soil REE concentrations are shown for both the surface and subsurface depths.

Soil pH and soil redox (Eh) values were compared graphically (Fig. 6) to show how the different samples distributed among the bauxite deposit groups. Some higher elevation samples, particularly the Los Arroyos, Avila, and Altagracia deposits, exhibited lower pH and higher Eh

values. A line drawn at pH 6 indicates the suggested threshold between REEs existing as cations (pH < 6) in soils, while soils beyond the threshold (pH > 6) point to continued REE complexation with solid-phase components (Villanova-de-Benavent et al., 2023).

Fig 6. Plot showing the relationship between soil pH and soil redox potential for the different bauxite deposits.

Calibration models for predicting soil REE concentrations

The Sierra de Bahoruco exists in a remote corner of the Dominican Republic, along the southwestern border with Haiti. Here, conducting the geochemical exploration in the traditional sense of employing a dedicated laboratory was challenging as logistical, infrastructural, and technical challenges abound in this area. It is possible to transport samples to developed regions of the country, such as the capital, Santo Domingo; however, when this study was executed, the capital represented a minimum six-hour drive, partially occurring on rough mountain roads. In addition, the sheer number of samples required to explore the bauxite deposits would rapidly enhance the operational expense and uncertainty of the campaign. Thus, opting to focus on a remote exploration scenario, we studied different approaches to measuring the REE concentrations in this expeditionary area.

Prediction plots showed the particular diagnostic features associated with the different calibration models (Table 1). The CoDA-based bulk composition (which does not include the measured soil REE concentrations) generated a highly accurate (R^2 = 0.987) prediction of total soil REE concentrations (Fig. 7A), with the ratio of predicted to observed values = 0.997. A highly accurate (R^2 = 0.975) calibration model was generated from the pXRF data (Fig. 7B). However, the limits of the pXRF model were apparent based on the separation of the model and the pure prediction

line. The model for pXRF data represented approx. 60% of the total REE concentrations (as determined by acid digestion). Furthermore, the current pXRF model could not reliably detect REE concentrations below 318 mg L^{-1} (the model's y-intercept), consistent with evidence related to XRF's difficulty in discriminating among the large-sized atoms. Nevertheless, this result greatly improves on previously published reliable detection limits for REEs of 1000 mg/kg (Simandl et al., 2014). The color sensor (Fig. 7C) generated a poor calibration model, representing < 47% of the total soil REE concentrations, with an estimated detection limit = 430 mg kg^{-1}.

Fig 7. Prediction plot showing the results of the ExtraTrees regression model showing the prediction of soil REEs based on (A) bulk composition, (B) pXRF analysis, and (C) NixPro2 color sensor data. The red dotted line and surrounding shading represents the extremely randomized trees regression function and its standard error correlating to the observed and predicted total REE concentration, with its fitted coefficient of determination or R^2. The black line represent the true diagonal of each prediction plot, indicating the ratio of observed to predicted values.

Table 1. Mean absolute error between the predicted and actual ∑REE (in ppm) for each composition.

Composition	Final Test set	Validation Set	Average Error
**Bulk**	50.5	114.9	174.1
**pXRF**	70.9	97.5	225.9
**Color**	74.5	301.1	265.9

Discussion

Viewed from a Soil Science perspective (Aide and Aide, 2012), the results point to differences in REE concentrations relative to the degree of material weathering and soil morphological development. Samples at higher elevations up the north side of the Sierra de Bahoruco, where the climate experienced greater precipitation, generally possessed higher quantities of REEs. In the face of widespread soils from a relatively homogenous origin (i.e., originating from long-term sustained weathering of volcanic ash material deposited across the area), then the higher soil REE concentrations were attributed to the greater, more progressive weathering environment that concentrated the elements in the soils as less resistant material dissolved away. In this case, the intense weathering experienced by the higher elevation bauxites was represented by the samples occurring below pH 6, representing free REE cations (i.e., outer-sphere complexes) released or "broken out" from their original or secondary mineral forms, such as carbonate-based complexes given the surrounding coral reef-derived limestone. Thus, as free cations, the REEs are expected to be easily extractable via a simple salt solution. Despite the overall highly weathered state of the bauxites, the lower elevation sites are expected to possess REEs that exists more as a mixture of outer- and inner-sphere complexes, requiring more aggressive extractants to harvest the elements.

A second feature of the data showed that REE concentrations increased with depth, where the soils typically exhibited a full clay texture. The development of an argillic subsurface horizon and the corresponding intensity of red color from an already high-clay soil represented evidence of the translocation of fine particles and the morphological development of the soil. Thus, it suggests that REEs exist as weak outer-sphere complexes and/or very fine mineral particulates in the soil translocated to the subsurface. Overall, the LREE:HREE ratio was > 1, consistent with

the literature regarding the nature of the hypothetical original pyroclastic material (Grant et al., 2005), enriched in elements like Nd, Pr, Ce, La, and Y. Interestingly, we note the slight decrease in the median LREE:HREE ratio toward 1 with depth for most of the samples, which is consistent with the scientific literature (Aide and Aide, 2012) showing that the ratio can substantially approach zero with depth. Thus, we hypothesize the presence of a "clay lens" within a specific span of the subsurface that may possess the most elevated concentrations at REEs due to natural soil development. The depth of this hypothetical clay lens, beyond the reach of our hand tools employed in this study, may partly explain the high REE concentrations in the Aceitilliar deposit group. As a formerly mined site, the actual depth of surface material removed was uncertain. From our observations in the field, the samples containing the highest concentrations of REEs and corresponding greatest shifts in the LREE:HREE ratio toward 1 occurred where we estimated approx. Previous mining activities removed 3-6 m of surface material. In contrast, most of the measured soil REE concentrations and its corresponding LREE:HREE ratios at the highly disturbed Las Mercedes mine site were "unremarkable" compared to the other sites, given that the samples were obtained with some 18 m of surface material removed, which we theorize is beyond the high REE clay lens. The exception here is represented by one sample taken at the Las Mercedes site from an area just above the deepest portion of the pit, perhaps 25 m in depth, by the side of the road cut, where the concentration of REEs reached approx. 1000 mg kg^{-1} on some of the analytical replicates. Further supporting evidence for the connections between REE concentrations and soil development originates from the high accuracy of the calibration model based on the development of the bulk composition from the soil characterization data. Typically viewed as "incompatibles" or indifferent ions in natural systems, REE concentrations were not expected to be influenced by the soil characterization data. However, the models predicted that

total REE concentration was highly predictable by the soil constituents. Thus, viewing REE concentrations and distributions relative to soil developmental perspective bore fruits in terms of connecting the long-term factors driving the weathering and morphological features of the local soils.

We point out here a critical limitation of this study related to the direct determination of REEs in the soils. While some papers utilize an acid-extraction technique (Khawassek et al., 2018; Lallemand et al., 2022; Reid et al., 2017; Villanova-de-Benavent et al., 2017), previous studies published by authors at the University of Barcelona (Torró et al., 2017; Villanova-de-Benavent et al., 2023) determined total soil REEs using an alkaline digestion technique for its ability to digest the REE-bearing minerals shown to exist on the site. While such an endeavor was beyond our capabilities for this particular study, we note that the soil REE concentrations were comparable to those published previously. This suggests that the acid-extraction approach showed the REEs existing in a high degree of outer-sphere complexation and, thus, operationally relevant. Subsequent studies involving differential extractions between acid- and alkaline-catalyzed digestions, sequential extraction extractions (Mittermüller et al., 2016), and solid-phase analysis, such as Synchrotron-based μ-XRF mapping and μ-x-ray diffraction (XRD) may help resolve these details – the latter perhaps being more efficient than the harvesting of microscopic quantities of REE-bearing minerals by intense separation methods.

Where the economic feasibility of exploiting such a resource is limited by the REE concentration of the deposit, mining material beyond this hypothetical clay lens may cross the boundaries of that threshold. Thus, a portion of the unique ecosystem in the otherwise impoverished Sierra de Bahoruco area may be spared once the hypothetical clay lens has been breached to minimize the overall environmental impact. Furthermore, the fact that the deposits

are located within 20 km of the ocean provides an opportunity for a potentially inexpensive seawater-derived extractant to remove REEs existing as weak outer-sphere (or exchangeable) complexes in the soil. For samples requiring a more aggressive extraction, such as acid, the abundant presence of limestone surrounding every deposit provides an opportunity for onsite neutralization of the soils after acid extraction. However, if the bauxites are increasingly mixed with or intruded by the underlying limestone substratum, then greater quantities of acid will be required to extract the REEs, given the inherent calcite buffering. Thus, a concerted effort is necessary to investigate how the potential intrusions of calcite minerals into the bauxite soils can impact the production costs of extracting the REEs.

pXRF proved convenient in the remote exploration of REEs only after calibration by laboratory results, given the technology's inherent limitations (Bryan et al., 2015; Simandl et al., 2014; Thermo Scientific, 2012). While pXRF proved less expensive than transporting soil samples back to Santo Domingo for analysis, the limitations associated with residual soil moisture in non-uniform particle size were significant in the data quality. Thus, the samples needed to be removed from the site and processed at the satellite laboratory before pXRF analysis occurred. There may be better, more convenient approaches to use in expeditionary settings. The model from the color sensor, used commonly in our morphological determinations in the field, was poorly accurate despite our anecdotal observation of the connection between soil REE concentrations and soil color. It seems that this poor accuracy is connected to the fact that REEs respond in a range of visible and NIR wavelengths (Balaram, 2022). If adequately calibrated by laboratory studies on previously collected soils concerning soil moisture (such as using a pressure plate system), remote exploration may potentially occur *in-situ* and immediately onsite using a field-portable vis-NIR reflection probe and soil moisture probe.

Conclusions

Viewing the REE concentrations, LREE:HREE ratios, and their distributions at the different deposits from a soil developmental perspective was instructive in understanding the resource's state and properties in the Sierra de Bahorcuo region. Considering the current remoteness of the Pedernales area and the terrain in which many deposits lie in the Sierra de Bahorcuo region, expeditionary techniques and approaches for conducting the geochemical exploration of REEs are essential. Thus, pXRF and possibly other analytical techniques may be useful for pushing forward the exploration into even more remote areas, assuming the developed models are applied only to the bauxite material (which seemed overall relatively homogeneous in composition) and not any calcite materials, which in this latter case, would require new model development.

Acknowledgements

The use of trade, product, or firm names in this report is for descriptive purposes only and does not imply endorsement by the U.S. Government. The tests described and the resulting data presented herein, unless otherwise noted, were obtained from research conducted under the Environmental Quality Technology Program of the US Army Corps of Engineers by the U.S. Army Engineer Research and Development Center (ERDC). Permission was granted by the Chief of Engineers to publish this information. The findings of this report are not to be construed as an official Department of the Army position unless so designated by other authorized documents. This work was partially supported by the U.S. Department of State's Embassy Science Fellowship program. The authors express gratitude to Mr. Alexander (Ted) Bryant and Ms. Juanita Aguirre of the U.S. Embassy in Santo Domingo, U.S. Department of State for their facilitating bilateral

coordination between the Dominican Republic and United States governments. Also, the authors express their gratitude to Ramón Morrobel and José Rodriguez from Ministerio de energía y minas, República Dominicana, and Jesus Rodriguez from Servicio geológico nacional, República Dominicana.

Author Contributions

Conceptualization: Mark Chappell, Charles Andros, Yoko Slowey, Autumn Acree

Data Curation: Mark Chappell, Charles Andros, Harold Rojas, Autumn Acree, Yoko Slowey

Formal Analysis: Mark Chappell, Charles Andros

Funding Acquisition: Marcelo Salles

Investigation: Mark Chappell, Harold Rojas, Leopoldo Gonzalez, Christine Young, Paige Fowler, Wesley Rowland, Michelle Wynter

Methodology: Mark Chappell, Charles Andros, Yoko Slowey, Autumn Acree

Project Administration: Mark Chappell, Marcelo Salles

Supervision: Mark Chappell, Harold Rojas

Validation: Christine Young, Michelle Wynter

Visualization: Mark Chappell

Writing - Original Draft Preparation: Mark Chappell

Writing – Review & Editing: Charles Andros, Yoko Slowey, Autumn Acree.

References

Aide, M. T., and Aide, C. (2012). Rare Earth Elements: Their Importance in Understanding Soil Genesis. *ISRN Soil Science* **2012**, 783876.

Aitchison, J. (1986). "The statistical analysis of compositional data," Chapman Hall, Ltd, London.

Aitchison, J. (1999). Logratios and Natural Laws in Compositional Data Analysis. *Mathematical Geology* **31**, 563-580.

Balaram, V. (2022). Rare Earth Element Deposits: Sources, and Exploration Strategies. *Journal of the Geological Society of India* **98**, 1210-1216.

Borst, A. M., Smith, M. P., Finch, A. A., Estrade, G., Villanova-de-Benavent, C., Nason, P., Marquis, E., Horsburgh, N. J., Goodenough, K. M., Xu, C., Kynický, J., and Geraki, K. (2020). Adsorption of rare earth elements in regolith-hosted clay deposits. *Nature Communications* **11**, 4386.

Bryan, R. C., Richers, D., Anderson, H. T., Ackman, T., and Gray, T. (2015). "Study on the utilzation of portable hand-held XRF spectroscopy as a screening tool for rare earth elemetns in coal and coal waste products." Tetra Tech, Pittsburg, PA.

Chappell, M. A., LeMonte, J. J., McGrath, C. M., Karna, R., Styles, R., Miller, C., Miller, L. F., Waites, M., Middleton, M. A., Price, C. L., Chappell, C. G., Dozier, H. R., Abraham, A. N., Henslee, A. C., and Strelzoff, A. (2022). Predicting Langmuir Model Parameters for Tungsten Adsorption in Heterogeneous Soil “Types” Using Compositional Signatures. *Geoderma* **422**, 115924.

Chappell, M. A., Seiter-Moser, J. M., West, H. M., Durham, B. D., Porter, B. E., and Price, C. L. (2019a). Building a quantitative analogy from soil classification systems using different compositional datasets. *PLOS One* **14**, e0212214.

Chappell, M. A., Seiter, J. M., West, H. M., Miller, L. F., Negrete, M. E., LeMonte, J. J., Porter, B. E., Price, C. L., and Middleton, M. A. (2019b). Predicting 2,4-dintroanisole (DNAN) sorption on various soil “types” using different compositional datasets. *Geoderma* **356**, 113916.

Chen, T., and Guestrin, C. (2016). XGBoost: A Scalable Tree Boosting System. *In* "Proceedings of the 22nd ACM SIGKDD International Conference on Knowledge Discovery and Data Mining", pp. 785–794. Association for Computing Machinery, San Francisco, California, USA.

Dahle, J. T., and Arai, Y. (2015). Environmental Geochemistry of Cerium: Applications and Toxicology of Cerium Oxide Nanoparticles. *In* "International Journal of Environmental Research and Public Health", Vol. 12, pp. 1253-1278.

Escuder-Viruete, J., Joubert, M., Abad, M., Pérez-Valera, F., and Gabites, J. (2016). The basaltic volcanism of the Dumisseau Formation in the Sierra de Bahoruco, SW Dominican Republic: A record of the mantle plume-related magmatism of the Caribbean Large Igneous Province. *Lithos* **254**, 67-83.

Geurts, P., Ernst, D., and Wehenkel, L. (2006). Extremely randomized trees. *Machine Learning* **63**, 3-42.

Goldich, S. S. (1947). "Aluminous Lateritic Soil of the Sierra de Bahoruco Area, Dominican Republic, W. I," U.S. Government Printing Office.

Grant, C. N., Lalor, G. C., and Vutchkov, M. K. (2005). Comparison of bauxites from Jamaica, the Dominican Republic and Suriname. *Journal of Radioanalytical and Nuclear Chemistry* **266**, 385-388.

Hedrick, J. B. (2004). Rare earths in selected U.S. defense applications. *In* "40th Forum on the Geology of Industrial Materials", Bloomington, IN.

Hernández Suárez, M., Molina Pérez, D., Rodríguez-Rodríguez, E. M., Díaz Romero, C., Espinosa Borreguero, F., and Galindo-Villardón, P. (2016). The Compositional HJ-Biplot—A New Approach to Identifying the Links among Bioactive Compounds of Tomatoes. *International Journal of Molecular Sciences* **17**, 1828.

Hu, X., Schappel, D., Silva, C. M., and Terrani, K. A. (2020). Fabrication of yttrium hydride for high-temperature moderator application. *Journal of Nuclear Materials* **539**, 152335.

Khawassek, Y. M., Eliwa, A. A., Haggag, E. S. A., Omar, S. A., and Abdel-Wahab, S. M. (2018). Adsorption of rare earth elements by strong acid cation exchange resin thermodynamics, characteristics and kinetics. *SN Applied Sciences* **1**, 51.

Koppen, W. (1936). "The Geographical System of the Climate," Borntraeger, Berlin.

Lallemand, C., Ambrosi, J. P., Borschneck, D., Angeletti, B., Chaurand, P., Campos, A., Desmau, M., Fehlauer, T., Auffan, M., Labille, J., Roche, N., Poizat, L., Collin, B., Rose, J., and Levard, C. (2022). Potential of Ligand-Promoted Dissolution at Mild pH for the Selective Recovery of Rare Earth Elements in Bauxite Residues. *ACS Sustainable Chemistry & Engineering* **10**, 6942-6951.

Leal Filho, W., Kotter, R., Özuyar, P. G., Abubakar, I. R., Eustachio, J. H. P. P., and Matandirotya, N. R. (2023). Understanding Rare Earth Elements as Critical Raw Materials. *Sustainability* **15**, 1919.

LeBlanc, A. R., Kennedy, L. M., Liu, K.-b., and Lane, C. S. (2017). Linking hurricane landfalls, precipitation variability, fires, and vegetation response over the past millennium from analysis of coastal lagoon sediments, southwestern Dominican Republic. *Journal of Paleolimnology* **58**, 135-150.

Mancheri, N., Sundaresan, L., and Chandrashekar, S. (2013). "Dominating the world: China and the rare earth industry." National Institute of Advanced Studies, Bangalore, India.

McInnes, L., Healy, J., and Melville, J. (2018). UMAP: Uniform Manifold Approximation and Projection for Dimension Reduction. *ArXiv:1802.03426*.

Melville, J. (2020). uwot: The Manifold Approximation and Projection (UMAP) Method for Dimensionality Reduction.

Mittermüller, M., Saatz, J., and Daus, B. (2016). A sequential extraction procedure to evaluate the mobilization behavior of rare earth elements in soils and tailings materials. *Chemosphere* **147**, 155-162.

Palarea-Albaladejo, J., and Martin-Fernandez, J. A. (2015). zCompositions - R package for multivariate imputation of left-censored data under a compositional approach. *Chemom. Intell. Lab. Syst.* **143**, 85-96.

Pawlowsky-Glahn, V., and Buccianti, A., eds. (2011). "Compositional Data Analysis: Theory and Applications." John Wiley & Sons, Ltd.

R Development Team (2018). R: A language and environment for statistical computing. Vienna, Austria.

Reid, S., Tam, J., Yang, M., and Azimi, G. (2017). Technospheric Mining of Rare Earth Elements from Bauxite Residue (Red Mud): Process Optimization, Kinetic Investigation, and Microwave Pretreatment. *Scientific Reports* **7**, 15252.

Rendleman, M. C., Smith, B. J., Canahuate, G., Braun, T. A., Buatti, J. M., and Casavant, T. L. (2022). Representative random sampling: an empirical evaluation of a novel bin stratification method for model performance estimation. *Statistics and Computing* **32**, 101.

RStudio Team (2018). RStudio: Integrated Development for R. RStudio, Inc., Boston, MA.

Simandl, G. J., Stone, R. S., Paradis, S., Fajber, R., Reid, H. M., and Grattan, K. (2014). An assessment of a handheld X-ray fluorescence instrument for use in exploration and development with an emphasis on REEs and related specialty metals. *Mineralium Deposita* **49**, 999-1012.

Sprecher, B., Kleijn, R., and Kramer, G. J. (2014). Recycling Potential of Neodymium: The Case of Computer Hard Disk Drives. *Environmental Science & Technology* **48**, 9506-9513.

Taggart, J. R. (2006). MID-HOLOCENE CLIMATE CHANGE IN SOUTHWESTERN DOMINICAN REPUBLIC: PROXY EVIDENCE FROM STABLE ISOTOPE VALUES AND MOLLUSCAN INDICES. *In* "19th Annual Keck Symposium". Curran Associates, Inc., Amherst, Massachusetts.

Thermo Scientific (2012). "Application Note: Exploration and mining of rare earth elements (REE) using tube-based Thermo Scientific Portable XRF analyzers." Thermo Scienrtific, Boston, MA USA.

Torró, L., Proenza, J. A., Aiglsperger, T., Bover-Arnal, T., Villanova-de-Benavent, C., Rodríguez-García, D., Ramírez, A., Rodríguez, J., Mosquea, L. A., and Salas, R. (2017). Geological, geochemical and

mineralogical characteristics of REE-bearing Las Mercedes bauxite deposit, Dominican Republic. *Ore Geology Reviews* **89**, 114-131.

Trytek, A. (2019). "Materials, technologies, and constructions: Special purpose metals. ," Rep. No. Order No. 100/19. Publishing House of the Rzeszów University of Technology, Rzeszów, Poland.

van den Boogaart, G., Tolosana-Delgato, R., and Bren, M. (2020). compositions: Compositional Data Analysis.

Villanova-de-Benavent, C., Proenza, J., Torró i Abat, L., Aiglsperger, T., Garcia-Casco, A., Altamirano Morán, D., Ramírez, A., and Rodríguez, J. (2017). "Secondary REE-Minerals in the Karst Bauxites of the Bahoruco Peninsula (Dominican Republic)."

Villanova-de-Benavent, C., Proenza, J. A., Torró, L., Aiglsperger, T., Domènech, C., Domínguez-Carretero, D., Llovet, X., Suñer, P., Ramírez, A., and Rodríguez, J. (2023). REE ultra-rich karst bauxite deposits in the Pedernales Peninsula, Dominican Republic: Mineralogy of REE phosphates and carbonates. *Ore Geology Reviews* **157**, 105422.

Figure1

Click here to access/download;Figure;Fig1.png

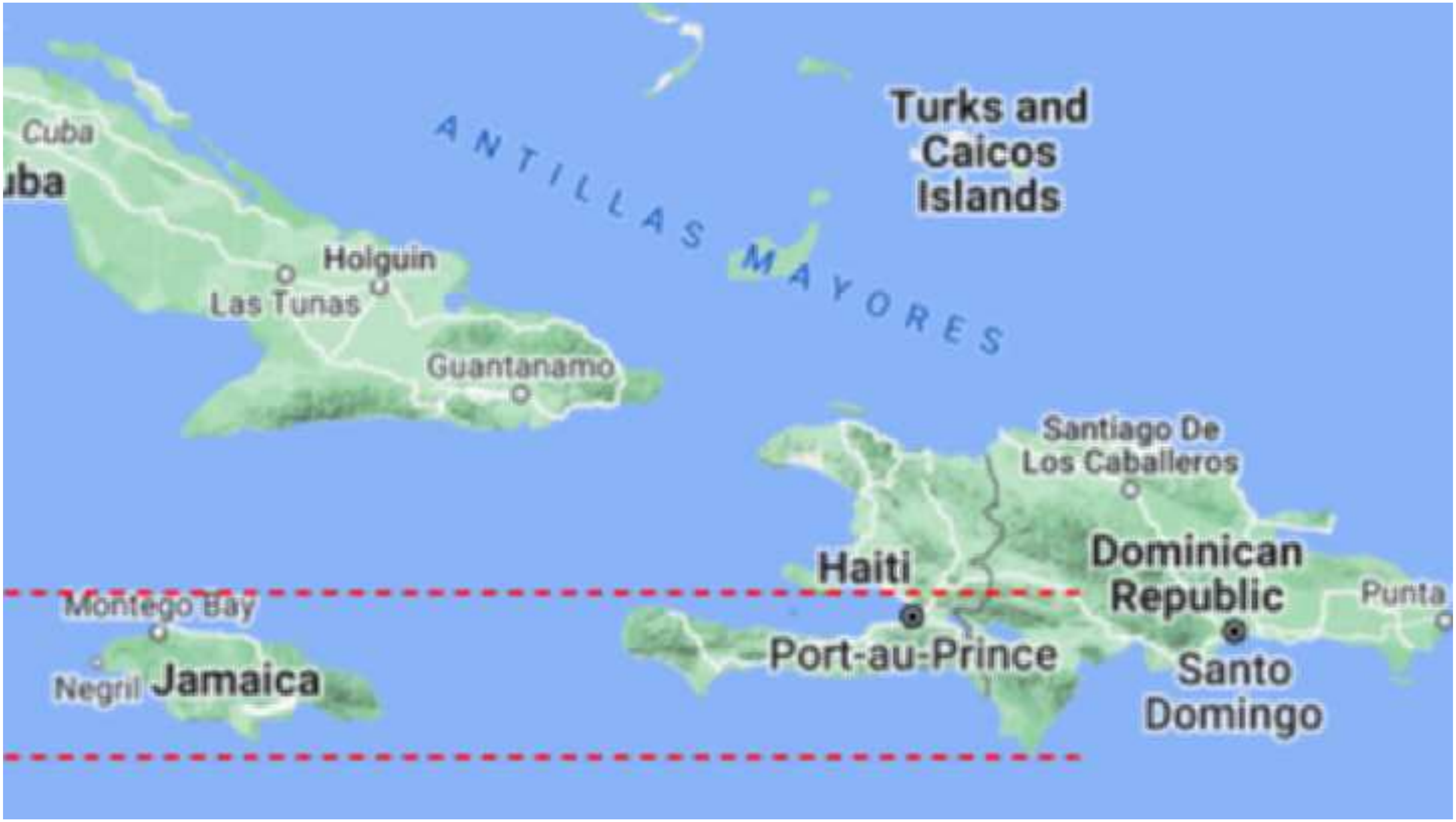

Figure2A

Click here to access/download;Figure;Fig2A.pdf

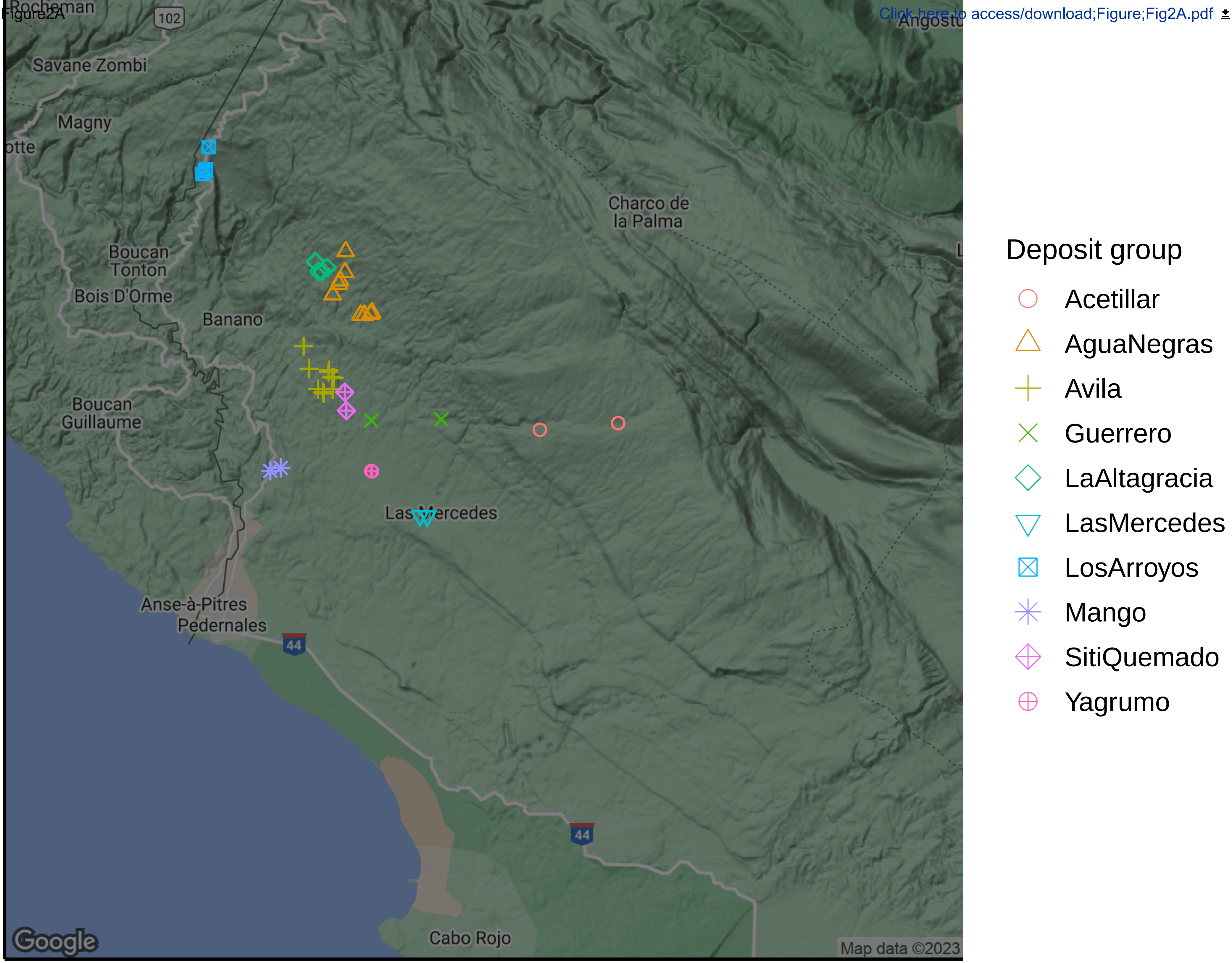

Figure2B

Click here to access/download;Figure;Fig2B.png

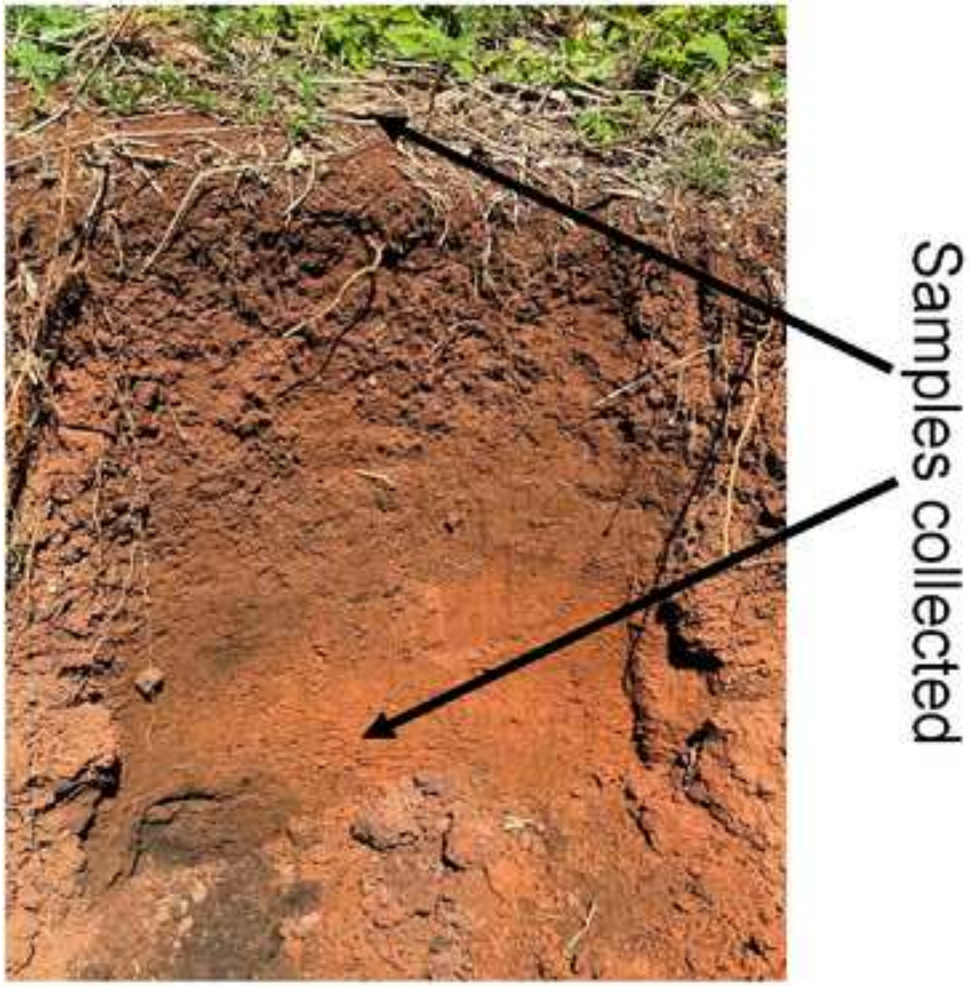

This figure is not well described in the results and discussion sections.

Figure3A

Click here to access/download;Figure;Fig3A.pdf

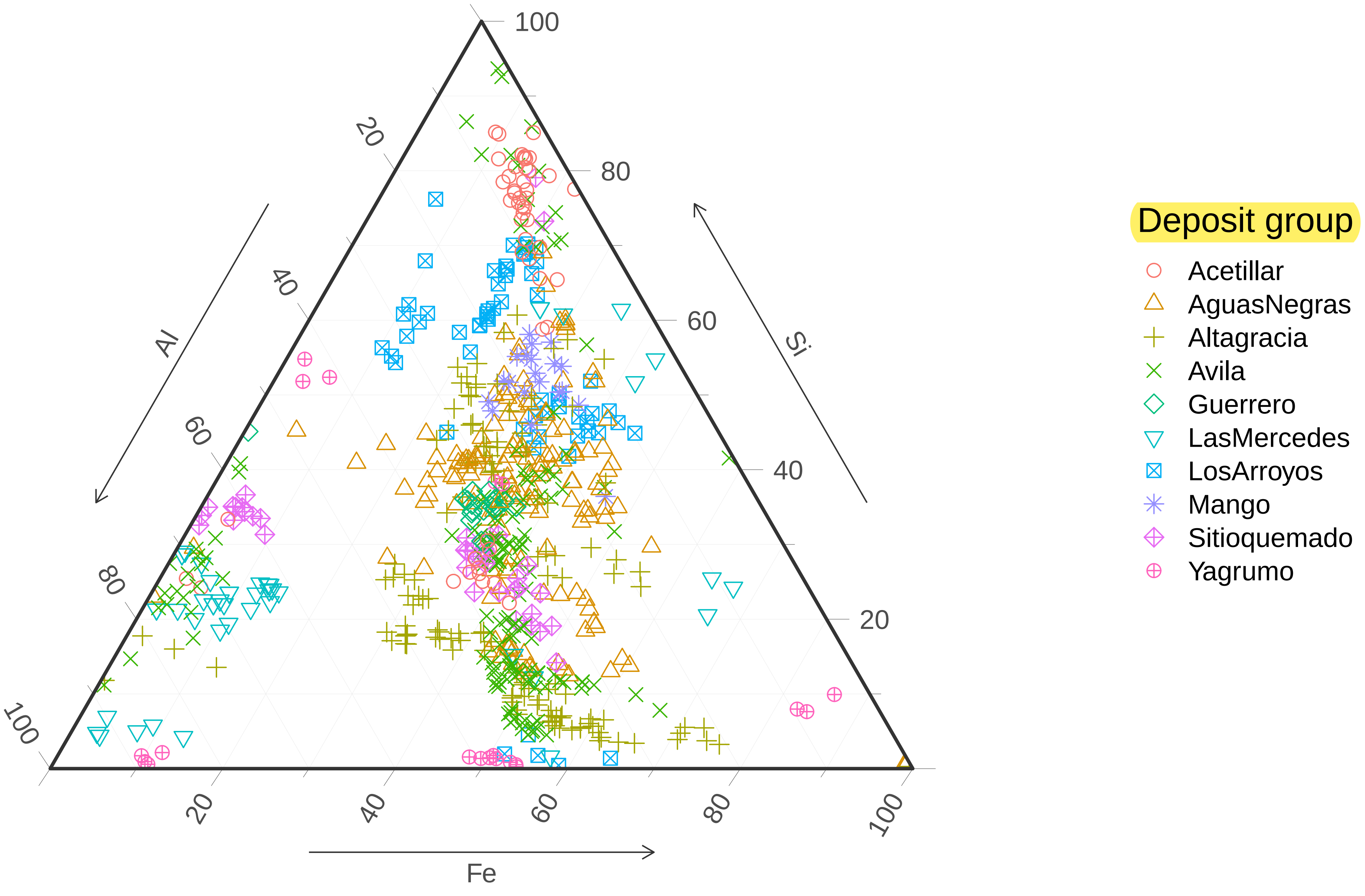

 Click here to access/download;Figure;Fig3B.pdf

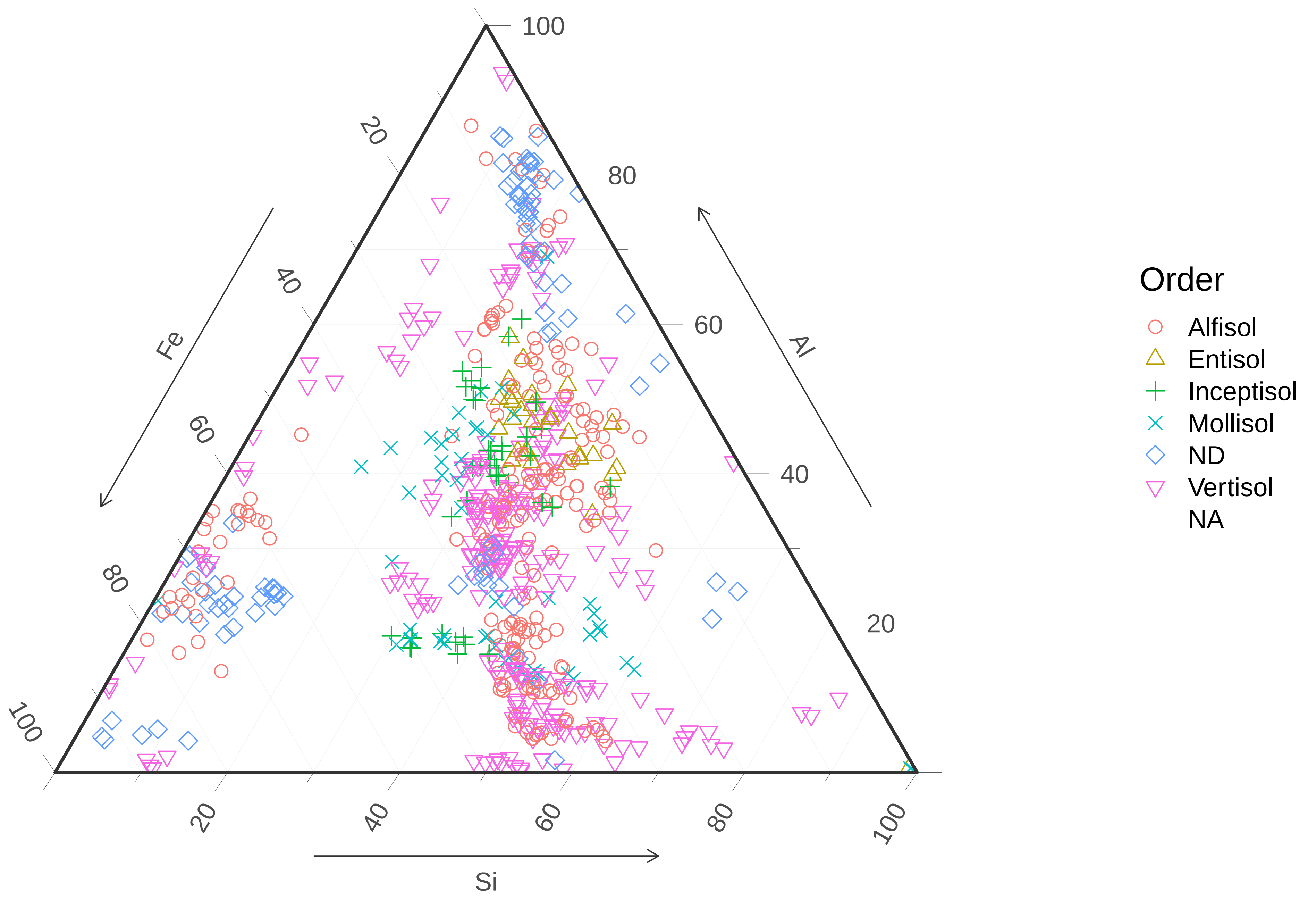

Figure3C

Click here to access/download;Figure;Fig3C.pdf

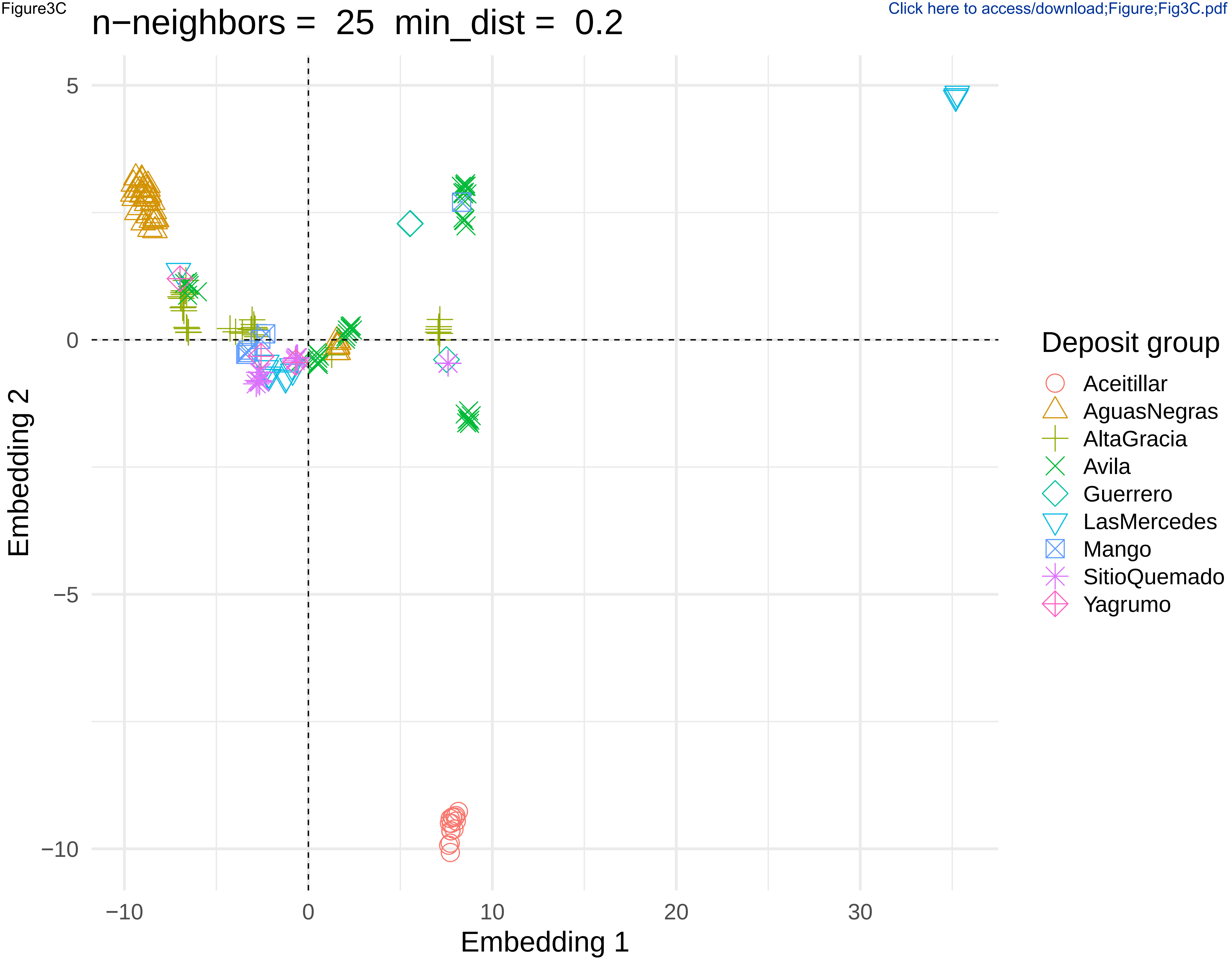

Figure4A

Click here to access/download;Figure;Fig4A.pdf

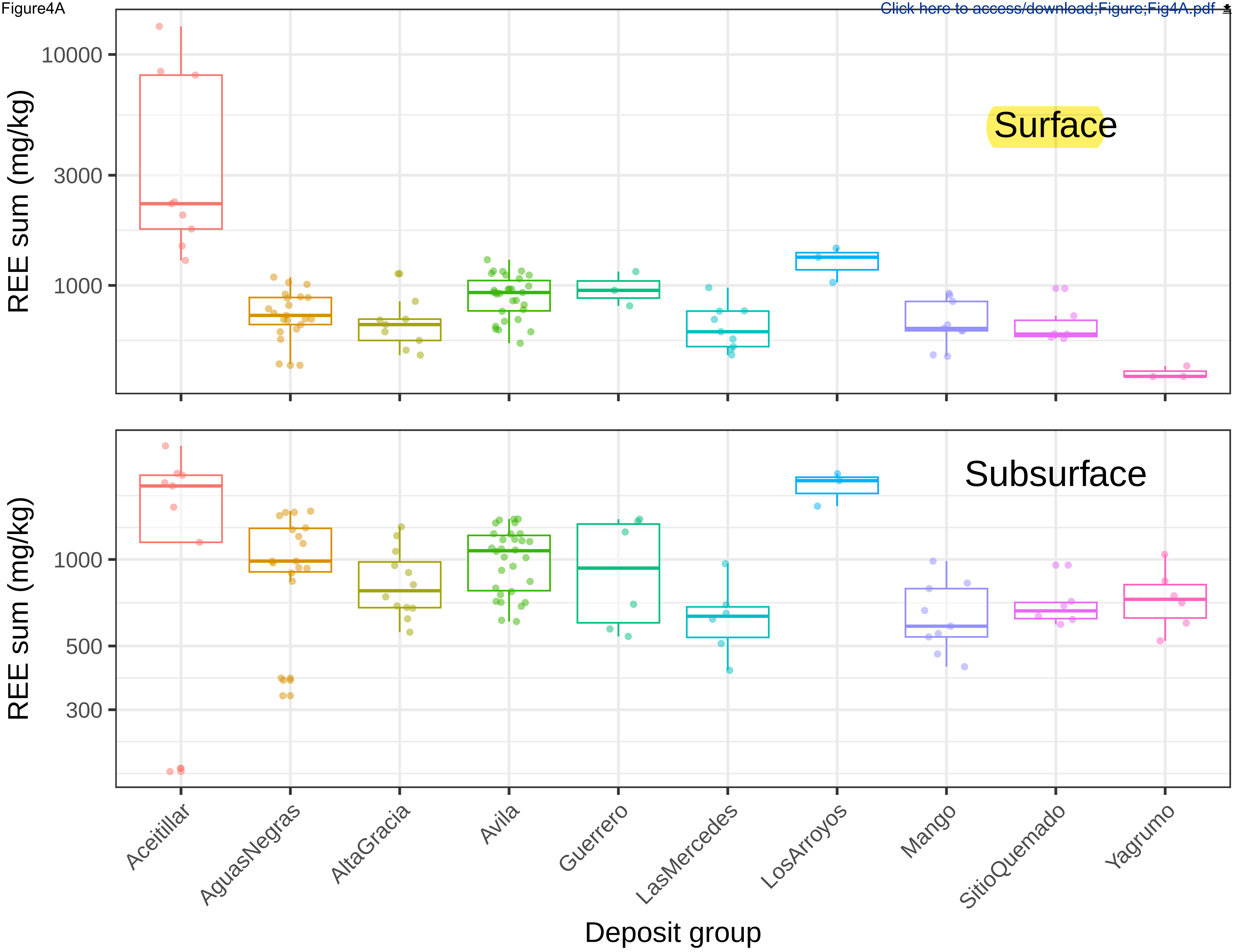

Figure4B

Click here to access/download;Figure;Fig4B.pdf

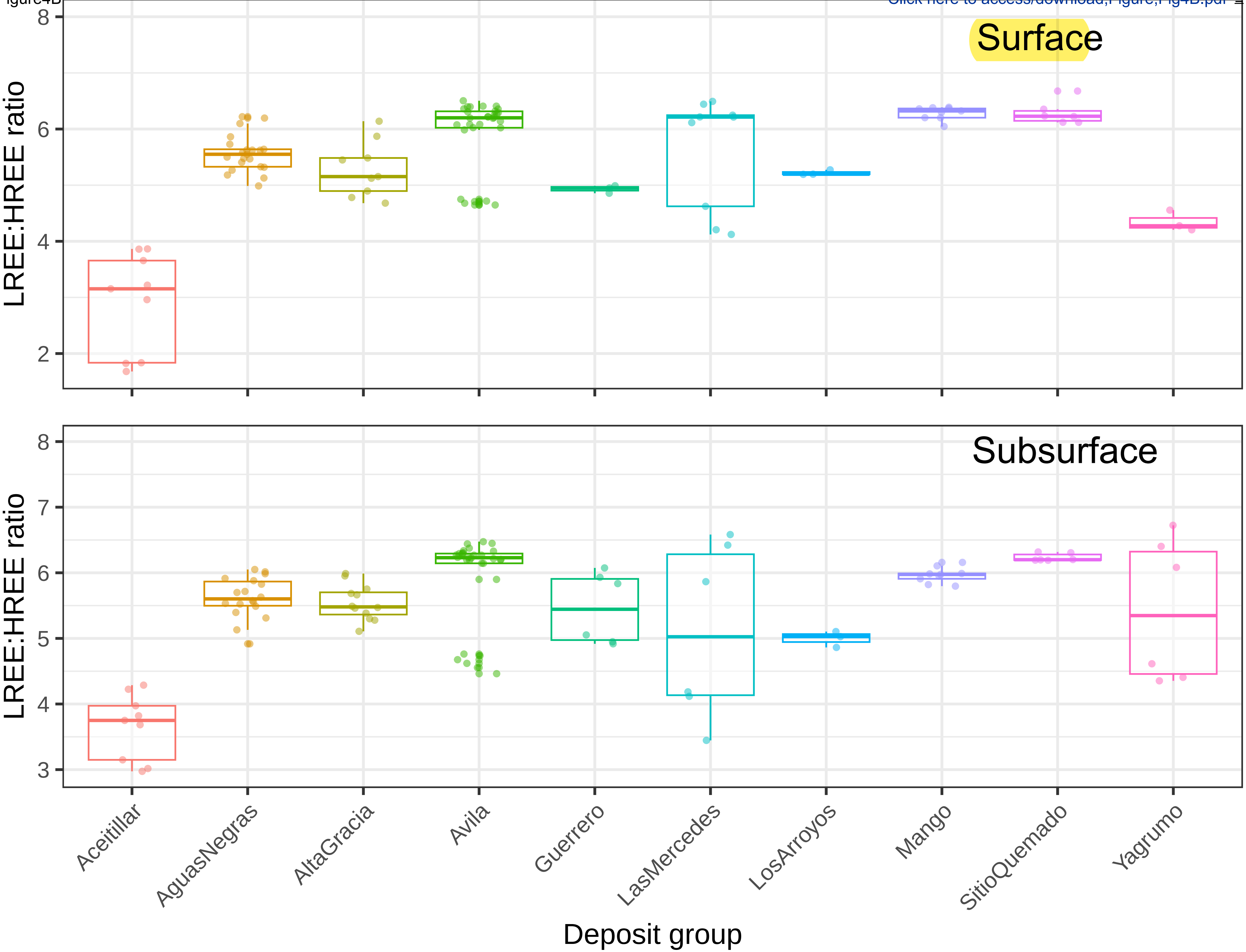

Figure5

Click here to access/download;Figure;Fig5.pdf

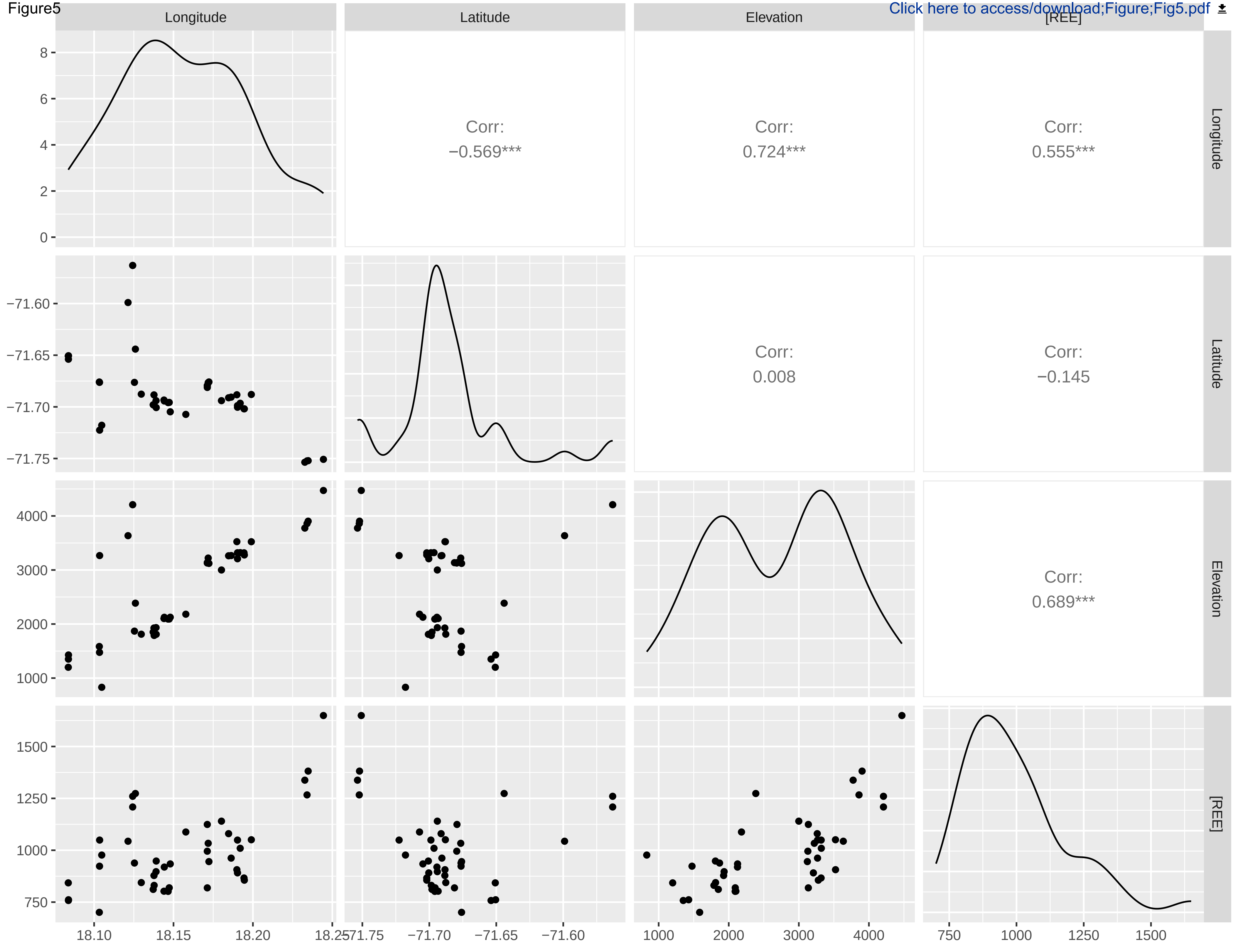

Figure6

Click here to access/download;Figure;Fig6.pdf

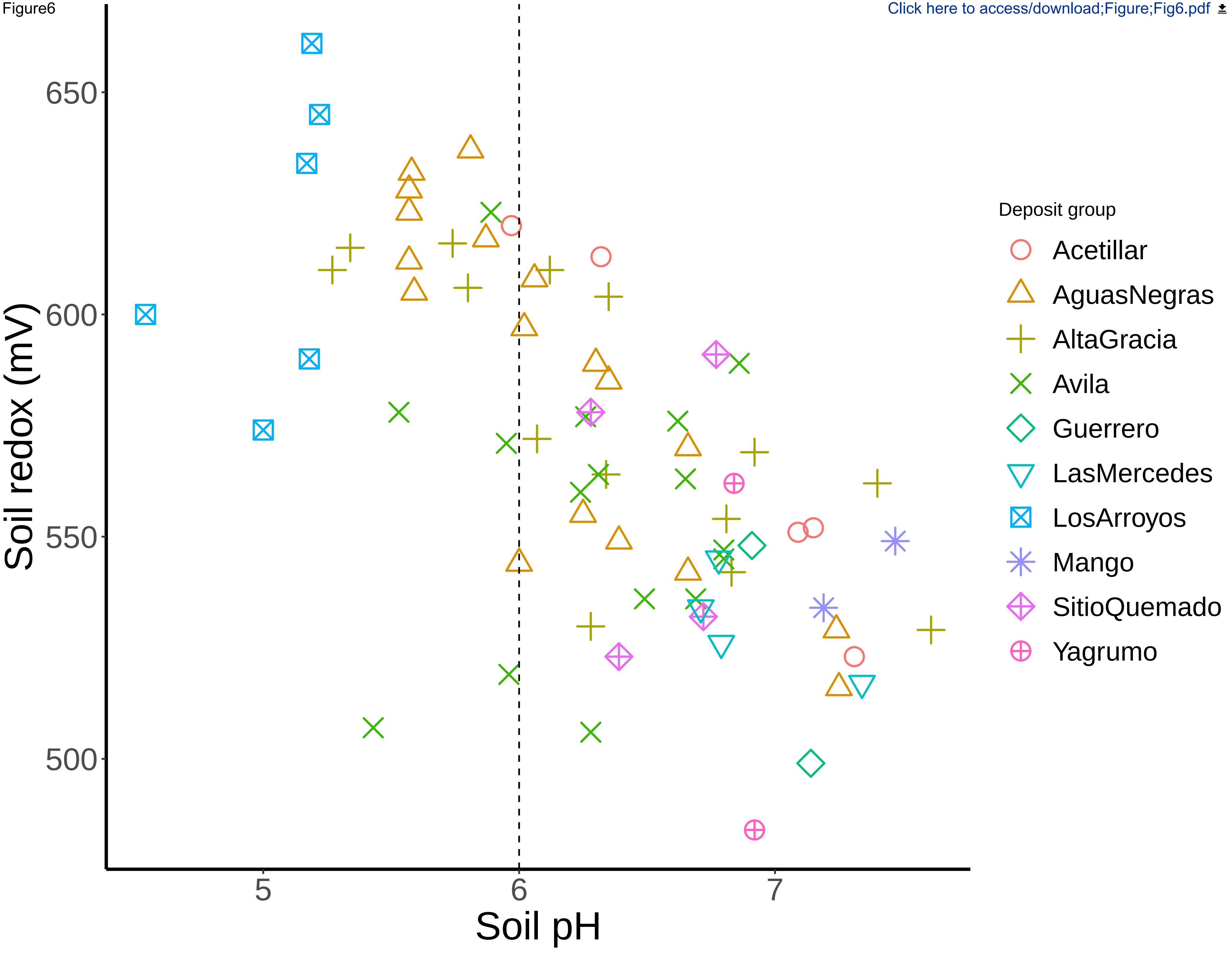

Figure7

Click here to access/download;Figure;Fig7.pdf

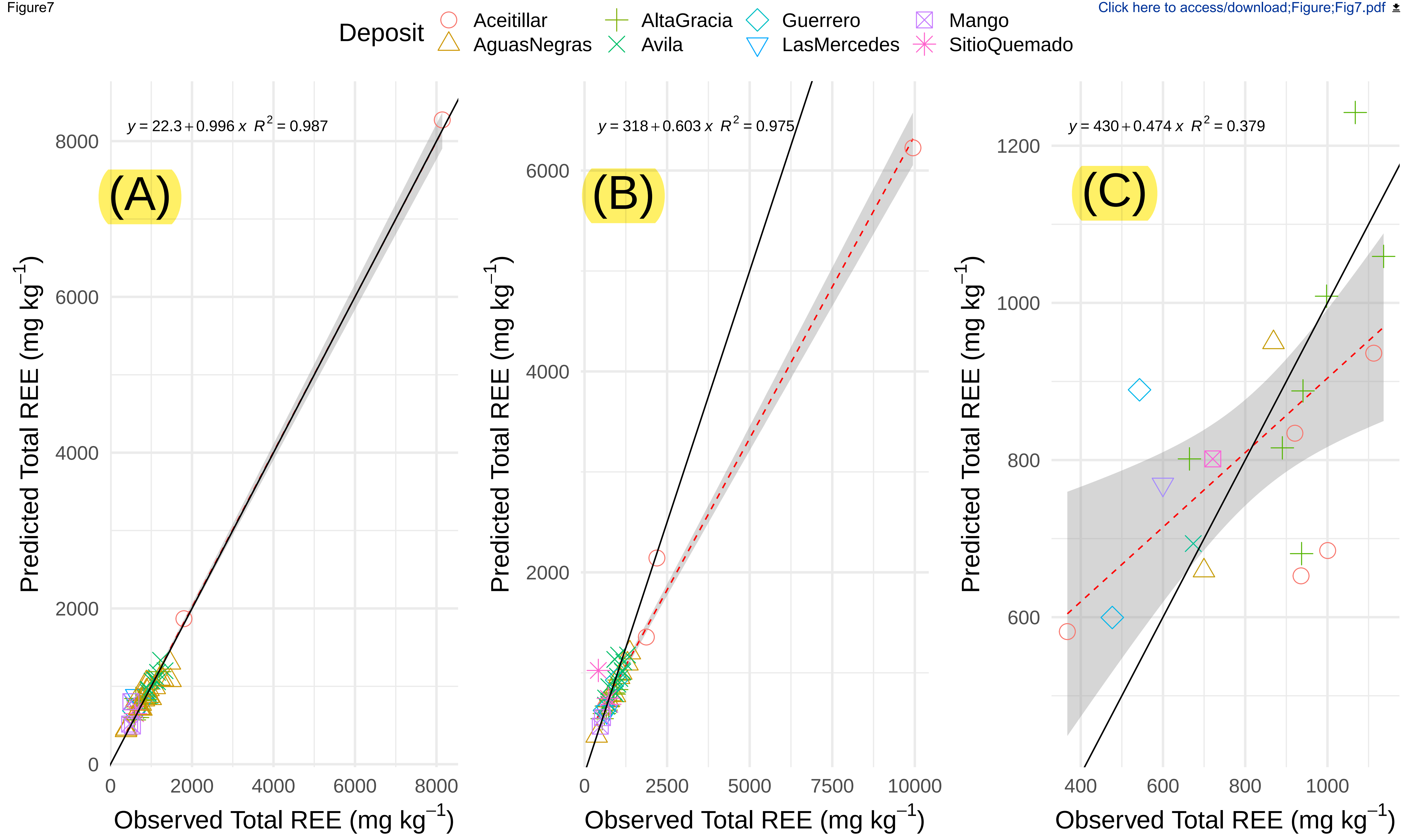

Supplement: Attachment — Submitted filename: PONE-D-23-43088_reviewer-completed.pdf [file pone.0315147.s004.pdf]
