# Supplementary material for: Geochemical exploration of rare earth element resources in highland karstic bauxite deposits in the Sierra de Bahoruco, Pedernales Province, Southwestern Dominican Republic
Source: PLoS One. 2025 Jan 10;20(1):e0315147. doi: 10.1371/journal.pone.0315147 (PMC11723596; doi:10.1371/journal.pone.0315147)
Supplement: Attachment — Submitted filename: Responses to Reviewers_revision2.1.docx [file pone.0315147.s006.docx]

Response to Reviewers (2^nd^ revision)

The changes made in this manuscript were in response to the comments made by the Editor and Reviewer 2, related to create subheadings for the Abstract section. In addition, Reviewer 2 requested that the Conclusions section of the manuscript include a research problem for future researchers.

Throughout this revision, we made minor changes to the manuscript as an additional careful review showed some inconsistencies in our explanations (held over from previous revisions), such as older descriptions of the models before changing to representing the error by RMSE values, and the inclusion of the resampling exercise. In particular, improvements in the ML modeling made the pXRF and color sensor comparable in terms of their prediction accuracies – a significant result now included in the Discussion section given the 1000x cost difference between the two sensors. In addition, we beefed up some of the discussion based on some of the newer literature about karstic bauxites in the area as well as potential insights from the scientific literature regarding Fe-oxyhyroxy – REE speciation that may influence their efficient extraction and recovery from the bauxites.

From these revisions, a higher-priority research question (the question itself requested by Reviewer2) emerged as the need for an efficient way to predict REE speciation (in addition to concentration, the other important component driving the economic feasibility of a resource) based on the output from the portable sensors. Extensive and geochemical speciation is applied typically to data developed under controlled laboratory conditions, but rarely if ever applied “retroactively” to the portable sensor responses. This, in part may be due to the general sense of the inherent error associated with these portable sensors, which we largely overcame computationally. For many heavy metal “contaminants”, the literature shows that soil speciation is often driven by the combination of concentration and the chemistry of a particular soil “type”. With REE concentrations exceeding 0.1 % in many cases within this deposit, the distribution of REE species may indeed be diverse and concentration-dependent.

In this paper, we also included the new figure containing the preliminary vis-NIR spectra for one of the bauxite samples. This figure serves two purposes: (i) helps to explain chemically REE predictability based on the color sensor and (ii) the potential value of expanding the spectral analytical range into the vis-NIR.
