# Supplementary material for: Geochemical exploration of rare earth element resources in highland karstic bauxite deposits in the Sierra de Bahoruco, Pedernales Province, Southwestern Dominican Republic
Source: PLoS One. 2025 Jan 10;20(1):e0315147. doi: 10.1371/journal.pone.0315147 (PMC11723596; doi:10.1371/journal.pone.0315147)
Supplement: Attachment — Submitted filename: Responses to Reviewers_revision3.docx [file pone.0315147.s007.docx]

Response to Reviewers (3rd revision)

The changes made in this manuscript were in response to the comments made by the Publisher to go back and remove the Abstract subheadings previously recommended by the Editor and Reviewer 2.
