# Supplementary material for: Geochemical exploration of rare earth element resources in highland karstic bauxite deposits in the Sierra de Bahoruco, Pedernales Province, Southwestern Dominican Republic
Source: PLoS One. 2025 Jan 10;20(1):e0315147. doi: 10.1371/journal.pone.0315147 (PMC11723596; doi:10.1371/journal.pone.0315147)
Supplement: Attachment — Submitted filename: Response to reviewer comments _REE_paper3.docx [file pone.0315147.s005.docx]

Dear PLosOne,

Enclosed is the revised manuscript, “Geochemical exploration of rare earth element resources in highland karstic bauxite deposits in the Sierra de Bahoruco, Pedernales Province, Southwestern Dominican Republic”. We appreciate the anonymous reviews provided for this manuscript. The reviewers’ questions at times were challenging, but their feedback instrumental in substantially improving the manuscript. In some cases, reviewers graciously provided additional references and even text to help clarify the underlying context of the manuscript. As a result, the entire manuscript was revised and improved based on the reviewers’ questions and comments.

Below are responses to reviewers’ comments and questions. Afterward, we included responses to the Editor’s questions.

**Responses to Reviewer #1**

1. The manuscript writing was closely reviewed and revised as suggested by the reviewer for greater clarity.
2. Done.
3. There seems to be a mixture of presenting the ratio between LREE and HREE with a “:” instead of a “/”. Thanks for pointing that out. Corrections were made throughout the manuscript.
4. Done.
5. There is only one list of REE that breaks them up into light and heavy categories, existing on L. 314-315 of the original manuscript. In that list, Y and Sc are both listed as light REE. L. 134 represents the beginning of the field measurement section.
6. We agree that representing REE concentration can be useful when expressed generally. However, the use of mg kg-1 in this manuscript represents an explicit unit of measurement by which the REE concentrations were determined. Specifically, it is unambiguous regarding how the REE mass was normalized. In other REE exploration studies, measurements of both sediments and waters exist, where unambiguous representation of REE concentration is also required. Stating our concentrations in units of mg kg-1 allows us to avoid any ambiguity in REE representation, and demonstrates that additional sampling “modes “are available for conducting the exploration.
7. Done
8. We appreciate the helpful introductory paragraph on bauxites provided by the reviewer and have incorporated it into the text.
9. The Conclusions section was expanded with a more extensive synopsis of the study.

**Responses to Reviewer #2**

L. 15. The abstract possesses the PLOS recommended 300-word limit.

L. 21. Respectfully, we disagree. This hypothesis refers to one of the more important themes emphasized throughout the manuscript. This investigation is not only researching the viability of pXRF for capturing the full range of REE measurements in the field (essentially improving upon the intrinsic capabilities of the instrument, especially overcoming limitations associated with quantifying the total of all 15 REEs), but the research is also focused on utilizing the geochemical information to geolocate the direction and orientation where we expect the REE concentrations to be the most elevated. Thus, as the title of the manuscript implies, REE distributions track the weathering intensity-driven developmental or morphological cues (accessible via hand digging given the remoteness of the area) of the soils, both in terms of subsurface development and elevation-driven climatic shifts observed throughout the Sierra de Bahoruco Mountains. To be clear, we have yet to find any papers that view REE distributions concerning soil morphological developmental features, aside from acknowledging the different regional soil “types” that may be involved. Also, note that the abstract was revised to no longer contain the hypothesis statement.

L. 21. REE was used consistently throughout the manuscript.

L. 26. To reduce the number of words in the Abstract as recommended by the reviewer, Fig. 2A has been revised to show the lat-lon coordinates of the site. Also, the coordinates were added to a new table in the revised manuscript (see below).

L. 41. Fixed the redundant definition of the REE abbreviation.

L. 132. We are unclear as to why the reviewer considers the Introduction section “too long”. Assuming 2.5 to 3 manuscript pages per printed page of PLoS One, we estimate that the Introduction section in the manuscript would occupy < 1.5 printed pages in the printed article. If there is information that the reviewer views as irrelevant or unimportant, the reviewer did not point out any particular section of text. Nonetheless, we attempted to make the Introduction section more concise. Note that this comment contradicts another reviewer’s comments to add a more detailed geological section. We did our best to reconcile the different requests.

L. 134. Following the reviewer’s recommendation, a table was added (now Table 1) that lists samples by their bauxite deposit group and their corresponding geospatial coordinates, elevation, depth of surface and subsurface samples, texture and color. This change was important to stay within the 300-word limit of the manuscript’s Abstract section.

L. 136. Done. See comment for L. 26.

L. 140. Text describing the observed soil profiles was included in the (i) revised caption for Fig. 2B and (ii) at the beginning of the Results section. The exact depths of the subsurface B horizons were not recorded given the difficulty of permeating the dense clay layer. Thus, we assume that the B horizons began at the limit of sampling.

L. 153. Citation added. Thanks!

L. 157. We weren’t sure how to interpret the reviewer’s comments here. I believe the reviewer is commenting that a color change in the profile does not represent a method for indicating increasing clay content – but not completely sure. Attempting to interpret the reviewer’s comments, our response is that this text does not refer to a method but in fact, is an observation consistently reported at these sites.

L. 161. Changed.

L. 163. All references in the revised text were reformatted to the PLoS format. Thanks!

L. 168. Citation added. Thanks, again!

L. 185. Changed.

L. 186. Added text noting that the proprietary standards refer to analytical standards included with the probe and meter.

L. 188. Company reference added. Thanks!

L. 197. It is difficult to discern the reviewer’s comment here. The previous sentence identified that the soil characterization data was transformed using CoDA to correct for geometric distortions in the data – a justification included in the text and explained with literature citations. The text starting on L. 197 describes the overall workflow associated with these geometric transformations, including data editing, zero imputation, and “closing” the compositions. These transformations represent precursor steps to the ML modeling. We added an introductory sentence that hopefully helps clarify this section further.

L. 218. Again, it is unclear what the reviewer is communicating here. The first sentence in the paragraph introduces UMAP as an effort to study the clustering of samples based on the geochemical information. There is no “scientific statement” per se, only a description of the method and workflow, which results are explained later in the manuscript. It was our hope that further partitioning out the modeling section with additional headers will help to alleviate the reviewer’s confusion.

L. 251. It is difficult to reconcile the reviewer’s complaint about the length of the Results section with the numerous requests for more detail. Thus, we endeavored to me more concise in our explanations.

L. 292. The relevant figure was referred to at the end of the sentence – Fig. 4A. We added “soil characterization” to be more specific about which data to be discussed in this section.

L. 392. To be clear, we intentionally separated the Results and Discussion sections instead of combining them into a single section. In our opinion, such a division makes it easier to discuss the data directly in the Results section, carefully going through each figure and table, comparing directly, point-for-point with the available scientific literature. Under this format, the Results section will be less speculative or more explicit to the immediate data at hand, which in this manuscript, occupied the previous 5 pages of text. Consequently, the Discussion section is more general, discussing information across the entire manuscript, containing broader interpretations and implicit representations of the overall message contained in the data. Thus, it seems that what the reviewers is interpreting as a non-specific Results section is actually the intended Discussion section, as outlined above.

L. 484. Done.

L. 495. Based on the PLoS One website, https://journals.plos.org/complexsystems/s/submission-guidelines#loc-author-contributions, “Those who contributed to the work but do not meet our authorship criteria should be listed in the Acknowledgments with a description of the contribution”. This section was edited to emphasize the contribution of those individuals listed. The remaining text represents a public disclaimer, and acknowledgment of the enterprise research and development programs in which this project was executed and is required by the U.S. government and ERDC to be included in all published work.

L. 511. Author contributions were expanded based on the reviewer's suggestion. Thanks.

Fig. 3A. This figure was not discussed in the Discussion section but in the Results section, starting on L. 255 in the previous version of the manuscript. We added some clarification regarding utilizing the breakout of the deposit groups in the ternary plots based on the bauxite definitions provided by Bardossy.

Fig. 4A and B. This figure represents a standard boxplot. A general description of the programming language and visualizations was provided on L. 176 of the revised manuscript.

Fig. 7 A-C is part of a multi-paneled plot. It is possible to combine plots 7A-C, but doing so may compromise the details of each individual plot, such as making the single plot so cumbersome that the diagonal, prediction line, corresponding confidence intervals, and equation are obscured.

**Responses to Reviewer #3.**

1. L. 204-205. Explanation was provided for our decision to conduct the transforms. Here, we clarified that the centered log ration (clr) transform was used for the bulk composition data while the additive log ratio (alr) was used for the pXRF data. The reasoning here is that the acid digestions used for generating the bulk composition don’t quantitatively consume Si. Thus, being unreliable, that column was removed before transforming the data. However, the pXRF does measure Si, so we normalized the data by Si as it is a common practice to do so for alumnosilicates. The reviewer is correct that the choice of transformation does influence the data; however, it was more important for us to remove the geometric distortion and get the correction. In our view, the choice of transformation method wasn’t all that important since the pXRF model was calibrated based on the total REE measured in the acid digest data, so it wasn’t necessarily important that the data columns line up between the bulk composition (which excluded the REE columns) and the pXRF data.

2. L. 219-221. More explanation was provided. We chose the approach for the zero interpolations as it seemed most reasonable based on the way the data is presented on the pXRF spectrometer. Ultimately, the choice of lower detection limits don’t matter all that much in the zero-interpolations, as that data contributes nominally to the overall signature. This is especially the case of the variable is largely comprised of NDs as it will most likely fall under the 5% our very conservative 5% missing data threshold, and will likely be removed before completing the zero interpolation anyway. But, we appreciate the question.

3. L. 2019-220. See the new Table 2 where the hyperparameters and initial estimates are listed. Also, see the revisions for a newly labeled section, called “ML regression modeling” as well as a new description of an optimized resampling scheme to show the potential best estimate of the model for the current dataset.

4. We replaced the MAE parameter with RMSE, with these values and the optimized R-squared parameters included in a new Table. 3.

5. A new Supporting Information document was created for this manuscript, which includes description and the plots from PCA and MDS analyses. These analyses were performed early in this work using the pXRF.

6. It seems that we did not exactly quantify this difference – it became obvious to us early in the work as the fewer REE were detected when the sample was anlayzed in the field vs. after processing in the lab. The outcome was sensible given our years of published Synchrotron-based x-ray work, which requires rigorous sample preparation. Additionally, those REE that were detected in the field were quantified at much lower concentrations when analyzed on the processed samples. Actually, we previously cited a publication in the original manuscript but for the revised manuscript, provided some additional explanation.

7. Unfortunately, we cannot as that data doesn’t exist. This was very much a proof of concept study, conducted on a shoe-string budget. However, thank you for the suggestion as we are commencing a much larger study beginning October 2024, and we can include lithological descriptors as a label to the new datasets.

**Responses to the Editor**

1. Based on the combined comments from the Editor and the three reviewers, the Introduction section was extensively revised. Some reviewers claimed the section was too long, others claimed that the Introduction required additional detail, such as requesting an added section detailing the areas geology, and a section written by one of the reviewers on bauxite. We did our best to reconcile these conflicting recommendations, attempting to make the Introduction section more concise, yet better focused on the specific information of interest to the reviewers.
2. See text added to the Introduction beginning on L. 50 of the revised manuscript which describes the geological setting of the study area described in this manuscript.
3. Done – see above response to the reviewer’s comment on L. 21.
4. Done. Extensive additions have been made to the manuscript casting greater detail on the ML modeling aspects. Also, see Table 3 in the revised manuscript.
5. Done, please see the expanded Discussion section.
6. Done, please see the expanded Conclusions section.
7. The manuscript has been extensively revised to better meet these requirements.
